# A Wilcoxon–Mann–Whitney Test for Latent Variables

**DOI:** 10.3389/fpsyg.2021.754898

**Published:** 2021-11-15

**Authors:** Heidelinde Dehaene, Jan De Neve, Yves Rosseel

**Affiliations:** Department of Data Analysis, Ghent University, Ghent, Belgium

**Keywords:** rank test, measurement error, indicators, robustness, nonparametric inference, group comparison

## Abstract

We propose an extension of the Wilcoxon–Mann–Whitney test to compare two groups when the outcome variable is latent. We empirically demonstrate that the test can have superior power properties relative to tests based on Structural Equation Modeling for a variety of settings. In addition, several other advantages of the Wilcoxon–Mann–Whitney test are retained such as robustness to outliers and good small sample performance. We demonstrate the proposed methodology on a case study.

## 1. Introduction

**Figure 1:**
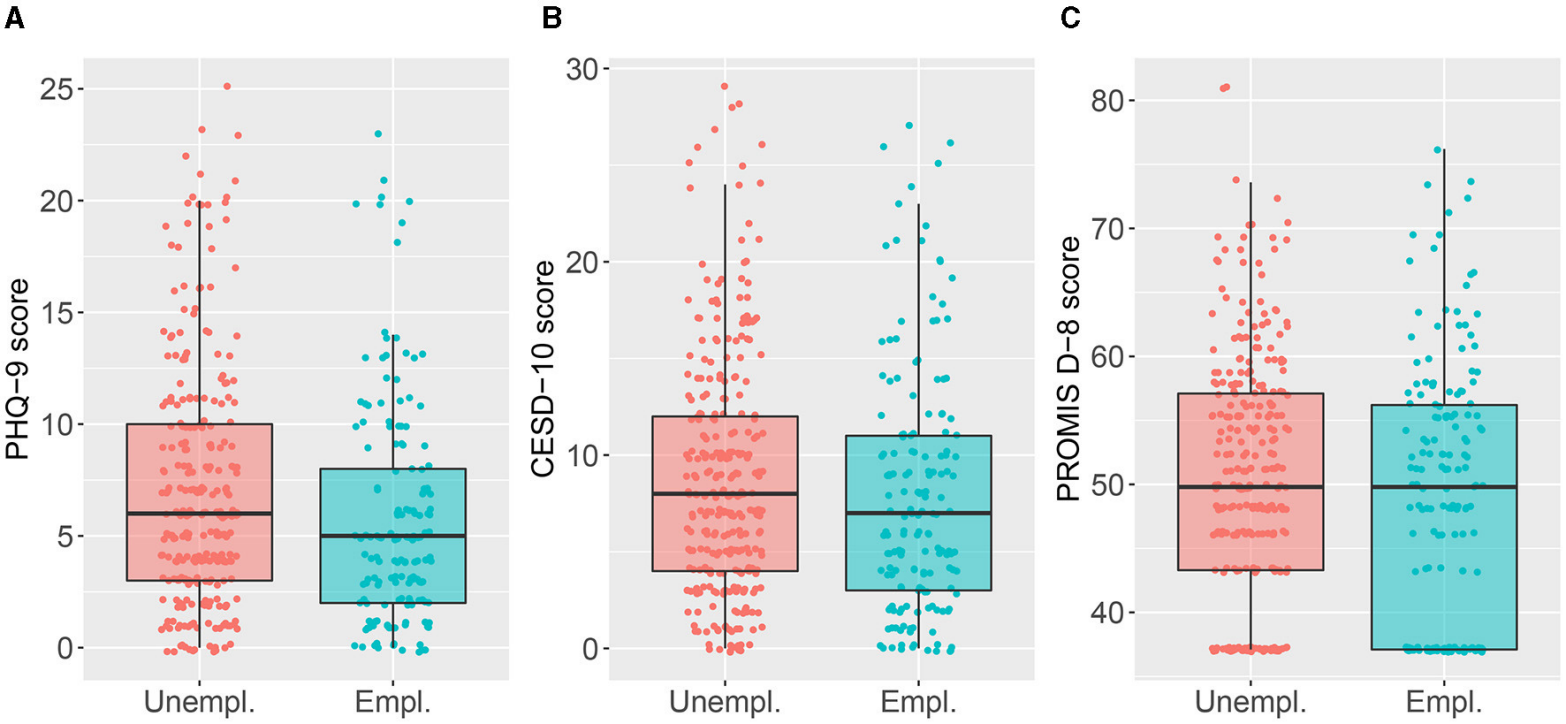
Boxplot of the scores for employed and unemployed subjects on three questionnaires measuring depression: **A**, the Patient Health Questionnaire-9 (PHQ-9, scale [0, 27]), **B**, the Center for Epidemiological Studies Depression Scale-10 (CESD-10, scale [0, 30]) and, **C**, the eight-item PROMIS Depression Short Form (PROMIS D-8, scores reported on a standardized scale with mean 50 and standard deviation 10). Higher scores indicate more symptoms of depression.

Consider a study where the interest is in the association between employment (yes/no) and the construct depression. The latter is quantified by the score on three questionnaires: the Patient Health Questionnaire-9 (PHQ-9; Spitzer et al., 1999; Kroenke et al., 2001; Kroenke and Spitzer, 2002), the Center for Epidemiological Studies Depression Scale-10 (CESD-10; Andresen et al., 1994), and the eight-item PROMIS Depression Short Form (PROMIS D-8; 8b short form; Pilkonis et al., 2011). The data originate from Amtmann et al. (2014), where the psychometric properties of these questionnaires were examined given that they all aim to screen for high depressive symptoms or major depressive disorder (MDD). Figure 1 displays these scores for 455 employed and unemployed individuals living with multiple sclerosis (MS). Scores for the PHQ-9 can range from 0 to 27, for the CESD-10 from 0 to 30 and the scores for PROMIS D-8 are reported on a standardized scale with mean 50 and standard deviation 10.

At first sight, the Wilcoxon–Mann–Whitney (WMW) test seems an appropriate choice to compare these two groups given the skewness and presence of outliers depicted in Figure 1. For each questionnaire, a WMW test can be carried out and for the sake of illustration, the WMW test will be introduced below for the PHQ-9 scores. The test considers the null hypothesis claiming that the distribution of the PHQ-9 scores is the same for both groups against the alternative stating that the probability that a subject of the unemployed group has a higher PHQ-9 score as compared to a subject of the employed group is different from 50%. The test statistic reflects this probability, also denoted as the probabilistic index, and the sole use of ranks hereby results in robustness against outliers. Under the null hypothesis and in the absence of ties, the sampling distribution of the test statistic only depends on the sample sizes of both groups, hence resulting in a distribution-free test (Thas, 2010). The WMW test often has superior power compared with the *t*-test for heavy tailed distributions (Blair and Higgins, 1980). For an exponential distribution, for example, the *t*-test needs approximately three times more observations than the WMW test to attain the same power (Van der Vaart, 2000; Hollander et al., 2013). Albeit both the WMW and the *t*-test aim to compare two samples, they apply different hypotheses and according effect sizes (i.e. probabilistic index vs. comparison of means) hence leading to different test properties.

Although the WMW test seems to be an appropriate choice in the current example, there is a complication as the outcome of interest, depression, is a latent variable. Stated otherwise, it is a variable that is not directly observed, but rather theoretically postulated or empirically inferred from observed variables (i.e. the indicators or proxies). Switching to latent variables, one cannot directly apply the methods that were designed for observed variables, because a measurement model that connects the latent variable to observed variables has to be postulated. Typically, continuous data with latent variables are analyzed via Factor Analysis (FA) or Structural Equation Modeling (SEM). The classical SEM has an optimal performance with respect to hypothesis testing when data are multivariate normally distributed. Given the skewness of the data, it is our interest to study whether the WMW test can be used in the context of latent variables while maintaining the attractive properties mentioned before. Applying the WMW test naively on each questionnaire results in a significant difference between the two groups on the first and second questionnaire (*p* = 0.003 and *p* = 0.04 respectively) but not on the third questionnaire (*p* = 0.12), making it difficult to obtain a global conclusion whether there is a significant association between depression and employment or not. In addition, these *p*-values do not take the measurement error into account. To the best of our knowledge, an extension of the WMW that takes into account those two essential aspects (i.e. combining the information of multiple indicators where measurement error is inherent) is not available yet and therefore the objective of this paper is to extend the WMW test in the context of latent variables with the main focus on hypothesis testing. The paper is organized as follows. In Section 2 we propose a WMW test for latent variables. Section 3 empirically contrasts the new methodology with existing methods in different settings by the use of a simulation study. Section 4 presents a case study and Section 5 provides a conclusion and discussion.

## 2. Method

We first introduce a measurement model that relates the latent variable to multiple indicators (or proxies). We then formulate the hypotheses of interest and demonstrate how they can be tested.

### 2.1. Measurement Model

The measurement model that relates the latent variable η to a set of indicators *Y*_*p*_ (*p* = 1, …, *P*), is given by


(1) $$\begin{array}{l} Y_{p}=h_{p}\left(\eta \right)+\varepsilon_{p} \end{array}$$

where *h*_*p*_(·) denotes a strictly monotone function and ε_*p*_ denotes the measurement error. To distinguish between the two groups, we use the notation (*Y*_*p*_, η, ε_*p*_) for the first group and ($Y_{p}^{*},\eta^{*},\varepsilon_{p}^{*}$) for the second. We define the reliability of an indicator *Y*_*p*_ as the proportion of variance in the indicator *Y*_*p*_ that is not explained by the measurement error ε_*p*_ and thus reflecting the quality of that indicator (Nunnally and Bernstein, 1994; Kline, 2015). Specifically,


(2) $$\begin{array}{l} \text{Rel}\left(Y_{p}\right)=\frac{\text{var}\left(Y_{p}\right)-\text{var}\left(\varepsilon_{p}\right)}{\text{var}\left(Y_{p}\right)}. \end{array}$$

The variance of the measurement error ε_*p*_ can be replaced by its empirical counterpart in order to obtain an estimate of the reliability of an indicator. We further elaborate on this estimation in the subsequent subsection.

Similar to classic factor analysis and SEM, comparing the latent means between two groups by the use of indicators is only feasible when assuming measurement invariance. Hence, the function *h*_*p*_(·) is assumed to be equal for the two groups and this needs to be confirmed by using e.g. SEM. In contrast with FA and SEM, where *h*_*p*_(·) is assumed to be linear, we impose a less stringent specification, i.e. monotone without imposing linearity. Garcia-Marques et al. (2014) pointed out that a curvelinear trend between a latent variable and its indicator may exist in psychological research due to e.g. ceiling or floor effects. An example of a floor effect is observed by Amtmann et al. (2014) for the PROMIS D-8 questionnaire and hence portrayed in the right panel of Figure 1. This demonstrates that assuming linearity can sometimes be an incorrect representation of reality.

The focus in this paper does not lie on estimating the measurement model and will thus be treated as a nuisance, in contrast with classic FA and SEM.

### 2.2. Hypotheses and Testing Procedure

We are interested in testing the following hypotheses which are expressed in terms of the distribution of the latent variable η (i.e. *F*_η_):


(3) $$\begin{array}{l} H_{0}:F_{\eta }=F_{\eta^{*}}\;H_{A}:F_{\eta }\neq F_{\eta^{*}}. \end{array}$$

If η_*i*_ (*i* = 1, …, *m*) and $\eta_{j}^{*}$ (*j* = 1, …, *n*) would be observable, the WMW test statistic (in the absence of ties) is given by $\left(\hat{\pi }-0.5\right)/\sigma_{0}$ where $\sigma_{0}=\sqrt{\left(m+n+1\right)/\left(mn12\right)}$ and


(4) $$\begin{array}{l} \hat{\pi }=\frac{1}{mn}\overset{m}{\underset{i=1}{\sum }}\overset{n}{\underset{j=1}{\sum }}\text{I}\left(\eta_{i}<\eta_{j}^{*}\right) \end{array}$$

with I(·) the indicator function (Wilcoxon, 1945; Mann and Whitney, 1947). Here, $\hat{\pi }$ is an unbiased estimator for *P*(η < η^*^), i.e. the probability that a randomly selected subject from group 1 has a lower outcome than a randomly selected subject from group 2.

In practice, η_*i*_ and $\eta_{j}^{*}$ are unobservable and therefore Equation (4) can not be computed. Instead, we only observe *Y*_*pi*_ and $Y_{pj}^{*}$ which are related to η_*i*_ and $\eta_{j}^{*}$ via measurement model (1). Let WMW($Y_{p},Y_{p}^{*};\alpha$) denote the WMW test applied to the indicators *Y*_*p*_ and $Y_{p}^{*}$ and where α denotes the level of significance. Under location-shift, i.e. $Y_{p}\overset{d}{=}Y_{p}^{*}+\Delta$, WMW($Y_{p},Y_{p}^{*};\alpha$) is an unbiased test for $H_{0}:F_{Y_{p}}=F_{Y_{p}^{*}}$ vs. $H_{A}:F_{Y_{p}}\neq F_{Y_{p}^{*}}$, meaning that the rejection level does not exceed α when $F_{Y_{p}}=F_{Y_{p}^{*}}$ and that it is at least α when $F_{Y_{p}}\neq F_{Y_{p}^{*}}$ (Lehmann, 1951).

Under the model


(5) $$\begin{array}{l} Y_{p}=h_{p}\left(\eta \right)+\varepsilon_{p},\text{ and }Y_{p}^{*}=h_{p}\left(\eta^{*}\right)+\varepsilon_{p}^{*},\text{ with }\varepsilon_{p}\overset{d}{=}\varepsilon_{p}^{*}, \end{array}$$

WMW($Y_{p},Y_{p}^{*};\alpha$) is also an unbiased test for $H_{0}:F_{\eta }=F_{\eta^{*}}$ vs. $H_{A}:F_{\eta }\neq F_{\eta^{*}}$. Indeed, when assuming equal distributions for the measurement error over the two groups per indicator, it follows that when $F_{\eta }=F_{\eta^{*}}$ then $F_{Y_{p}}=F_{Y_{p}^{*}}$ so that the rejection level of WMW($Y_{p},Y_{p}^{*};\alpha$) does not exceed α when $F_{\eta }=F_{\eta^{*}}$. Secondly, when $F_{\eta }\neq F_{\eta^{*}}$ then $F_{Y_{p}}\neq F_{Y_{p}^{*}}$ so that the rejection level is at least α when $F_{\eta }\neq F_{\eta^{*}}$ and under the assumption of location-shift (i.e. $Y_{p}\overset{d}{=}Y_{p}^{*}+\Delta$). Consequently, the test statistic


(6) $$\begin{array}{l} \frac{{\left(mn\right)}^{-1}\overset{m}{\underset{i=1}{\sum }}\overset{n}{\underset{j=1}{\sum }}\text{I}\left(Y_{pi}<Y_{pj}^{*}\right)-0.5}{\sigma_{0}} \end{array}$$

results in an unbiased test for $H_{0}:F_{\eta }=F_{\eta^{*}}\text{vs.}H_{A}:F_{\eta }\neq F_{\eta^{*}}$. In summary, we can apply the WMW test on the observed data to test hypotheses concerning the latent variable. This testing procedure does not require that *h*_*p*_(·), var(ε_*p*_), var($\varepsilon_{p}^{*}$) have to be estimated nor that *h*_*p*_(·) needs to be linear. The strong assumption of equal distributions for the measurement error is required for the theoretical validation of this method. However, violations of this assumption will appear to have no detrimental consequences with respect to hypothesis testing in our simulation study (see Section 3).

When multiple indicators are available, we propose to aggregate them to obtain a new indicator where the above-mentioned test rationale still holds. This aggregated indicator can be superior in terms of its reliability in comparison with the original indicators, but it however requires estimates of the measurement error variance. The aggregated indicator is obtained by making a linear combination of the original indicators while preserving measurement invariance as mentioned in Section 2.1. Therefore, the construction of this linear combination is based on all data of an indicator *p*, i.e. both $Y_{p}\text{and}Y_{p}^{*}$. Let *Z*_*pk*_ denote an observation from the pooled sample *Y*_*p*_ and $Y_{p}^{*}$ where *k* = 1, …, (*m*+*n*). We define the aggregated indicator as


(7) $$\begin{array}{l} Y_{k}^{AGG}=\overset{P}{\underset{p=1}{\sum }}a_{p}Z_{pk}, \end{array}$$

where the weights a_*p*_ in Equation (7) can be chosen according to two strategies. A first strategy is to simplify the aggregation to an unweighted mean of the standardized indicators. However, treating all indicators as equally important is a reduction of the complexity in reality where some indicators are more reliable than others. Therefore, we propose a second strategy where the weights a_*p*_ in Equation (7) are chosen so that the estimated reliability of $Y_{k}^{AGG}$ is maximized (Bentler, 1968; Li, 1997; Penev and Raykov, 2006). In other words, a maximally reliable composite is constructed and the estimated reliability of $Y_{k}^{AGG}$ is by construction at least equal to the highest estimated reliability of the separate indicators used in the aggregation.

In order to obtain the weights *a*_*p*_, one needs to estimate the variance of the measurement error of the accompanying indicators, comprising the data of both groups. Given the data structure of our simulation study (Section 3) and case study (Section 4), we briefly elaborate on how this variance can be estimated when at least three indicators are available. For a more exhaustive explanation on how to estimate the variance of measurement error under different settings, we refer to De Neve and Dehaene (2021). Imposing a linear relationship among the indicators *Y*_1*i*_ = *h*(η_*i*_)+ε_1*i*_, *Y*_2*i*_ = *a*_2_+*b*_2_*h*(η_*i*_)+ε_2*i*_ and *Y*_3*i*_ = *a*_3_+*b*_3_*h*(η_*i*_)+ε_3*i*_, it follows that whenever cov(*Y*_1_, *Y*_2_) ≠0, cov(*Y*_1_, *Y*_3_) ≠0 and cov(*Y*_2_, *Y*_3_) ≠0 that var(ε_1_) = var(*Y*_1_) - cov(*Y*_1_, *Y*_2_)cov(*Y*_1_, *Y*_3_)/cov(*Y*_2_, *Y*_3_). Although the assumption of a linear relationship among the indicators is required for these formulas, the results of our simulation study show no detrimental consequences of violations.

The test rationale that has been put forward earlier on, i.e. employ the WMW test on observed data to make inference with respect to latent variables, is still valid when using this aggregated indicator $Y_{k}^{AGG}$. Moreover, this aggregation does not complicate the justification due to the flexibility of the measurement model, because the aggregated indicator can be rewritten as


$$\begin{array}{lll} Y_{k}^{AGG} & = & h^{AGG}\left(\eta_{k}\right)+\varepsilon_{k}^{AGG},\;h^{AGG}\left(\cdot \right)=\overset{P}{\underset{p=1}{\sum }}a_{p}h_{p}\left(\cdot \right),\\ \varepsilon_{k}^{AGG} & = & \overset{P}{\underset{p=1}{\sum }}a_{p}\varepsilon_{pk}, \end{array}$$

and hence the same structure as described in measurement model (1) still holds. Therefore, we obtain the following test statistic:


(8) $$\begin{array}{l} U^{AGG}=\frac{{\left(mn\right)}^{-1}\overset{m}{\underset{i=1}{\sum }}\overset{n}{\underset{j=1}{\sum }}\text{I}\left(Y_{i}^{AGG}<Y_{j}^{AGG}\right)-0.5}{\sigma_{0}}, \end{array}$$

where the data of the two groups for the aggregated indicator are distinguished by using the notation $Y_{i}^{AGG}$ and $Y_{j}^{AGG}$ for respectively the first and second group. Similar with the standard WMW test, a *p*-value can be obtained by either using a permutation null distribution or a standard normal approximation (Wilcoxon, 1945; Mann and Whitney, 1947; Thas, 2010).

### 2.3. Sample Size and Power Calculation

An approximate total sample size *N* (*N* = *m*+*n*) for a one-sided test with significance level α can be determined by using the formula from Hollander et al. (2013):


(9) $$\begin{array}{l} N=\frac{{\left(z_{\alpha }+z_{\beta }\right)}^{2}}{12c\left(1-c\right){\left(\delta -\frac{1}{2}\right)}^{2}}, \end{array}$$

where *c* reflects the ratio of the sample sizes of the two groups, i.e. $c=\frac{m}{m+n}$, and δ denotes the effect size under the alternative hypothesis, i.e. $P\left(Y_{1}^{AGG}<Y_{2}^{AGG}\right)$. Subsequently, the expected power can be deduced via


(10) $$\begin{array}{l} 1-\beta =\Phi \left(\sqrt{N12c\left(1-c\right){\left(\delta -\frac{1}{2}\right)}^{2}}-z_{\alpha }\right). \end{array}$$

## 3. Simulation Study

In order to assess the finite sample performance of the extended Wilcoxon–Mann–Whitney test, a simulation study is performed. All simulations and analyses are performed with R version 3.5.1 (R Core Team, 2020).

Different scenarios are explored, all based on the following data generating process:

**Latent variables**:


(11) $$\begin{array}{l} \begin{array}{cc} \eta & =\beta +\zeta \end{array}\;\begin{array}{cc} \eta^{*} & =\beta^{*}+\zeta^{*} \end{array} \end{array}$$

**Observed variables/indicators**:


(12) $$\begin{array}{l} \begin{array}{l} Y_{1}=\eta +\varepsilon_{1}\\ Y_{2}=h_{2}\left(\eta \right)+\varepsilon_{2}\\ Y_{3}=h_{3}\left(\eta \right)+\varepsilon_{3} \end{array}\;\begin{array}{l} Y_{1}^{*}=\eta^{*}+\varepsilon_{1}^{*}\\ Y_{2}^{*}=h_{2}\left(\eta^{*}\right)+\varepsilon_{2}^{*}\\ Y_{3}^{*}=h_{3}\left(\eta^{*}\right)+\varepsilon_{3}^{*} \end{array} \end{array}$$

**Figure 2:**
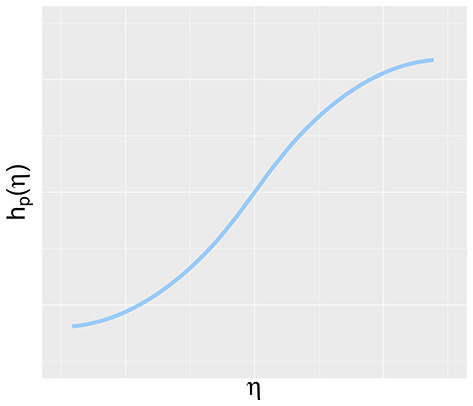
Illustration of the curvilinear trend between a latent variable η and the function *h*_*p*_(η) as used in the simulation study.

To explore the performance of the suggested methodology under different scenarios, the simulation study covers both normal, heavily tailed and skewed distributions for ζ and ζ^*^ (and consequently η and η^*^): N(0,1), *t*_5_, *Laplace*(0, 1.25) and the standard exponential centered around zero. In the wide range of possible non-normal distributions, these distributions correspond to kurtosis and skewness values that can be encountered in practice and that are leptokurtic (Chou et al., 1991). This positive kurtosis enables the examination of whether the superior power of the WMW test in heavier tailed distributions is carried over to the context of latent variables (Van der Vaart, 2000; Hollander et al., 2013). The superiority in heavier tailed distributions is also observed in small samples and therefore sample sizes in this simulation study are varied between 15, 50, and 100 observations in each group. As a result, exploration of the properties of different methods is possible without running into a ceiling effect with respect to the empirical power. By varying the variances of the measurement error, the influence of the reliability of the indicators on the different methods can be studied. We consider reliabilities of 60% and 80% which corresponds to indicators that can be considered as having a relatively weak and an adequate reliability respectively (Nunnally and Bernstein, 1994; Jackson, 2001). The function *h*_*p*_(·) is either linear or non-linear. In the non-linear case, the transformation *h*_*p*_(·) equals Φ^−1^[*F*(·)] where F equals the *t*-distribution with 1 or 3 degrees of freedom. This relationship can be seen as a modified inverse logit function and was chosen since it recreates a curvilinear trend between a latent variable and its indicator, in accordance with the trend mentioned in Garcia-Marques et al. (2014). Figure 2 shows an example of such a curvilinear trend between a latent variable η and *h*_*p*_(η).

Taking into account all parameters discussed up till now, the simulation study involves linear or non-linear functions *h*_*p*_(·), four error distributions, three sample sizes and two reliabilities, resulting in 48 simulation scenarios. For all these 48 combinations, four settings are considered to gain insight with respect to the empirical consequences of violating the assumption of equality in distribution of the measurement error as postulated under model (5) in Section 2.2.

- Setting 1: $\varepsilon_{p}\overset{d}{=}\varepsilon_{p}^{*}$, i.e. a correctly specified model.

In setting 2 and 3, the model is misspecified.

- Setting 2: $c\varepsilon_{p}\overset{d}{=}\varepsilon_{p}^{*}$ with *c*≠1, i.e. the variance of the measurement error differs across groups, where *c* is chosen in such a way that the reliability of all indicators in the second group is consistently about 5% lower than in group 1.
- Setting 3: $\varepsilon_{p}\overset{d}{\neq }\varepsilon_{p}^{*}$ but $\text{Var(}\varepsilon_{p})=\text{Var}(\varepsilon_{p}^{*}\text{)}$, i.e. the distribution of the measurement error differs across groups (a normal distribution and Laplace distribution respectively), but the variance is equal.

In the last setting, we consider a correctly specified model but with an indicator with very low reliability:

- Setting 4: $\varepsilon_{p}\overset{d}{=}\varepsilon_{p}^{*}$, *Y*_3_ and $Y_{3}^{*}$ have a reliability of only 20%.

In order to assess the impact of all these parameters on both the empirical Type I error rate and power, the parameter β^*^ as defined in Equation (11) is varied. The exact parameter value depends on the error distribution, but it is chosen so that the probabilistic index *P*(η < η^*^) equals 50% and 65% under the null and alternative hypotheses respectively. The rationale for this probabilistic index can be traced back to the simple relationship between a probabilistic index and the standardized difference as described in Cohen (1988) and De Schryver and De Neve (2019). By exploiting this relationship, a probabilistic index of 65% coincides with a standardized effect of 0.55 and is hence defined as a medium effect according to Cohen (1988).

For each setting, 1,000 Monte Carlo simulation runs are used to evaluate and contrast the performance of six methods. In the first method, further referred to as **WMW–max rel**, a maximally reliable composite is used as input for the WMW test. In this simulation study, the weights *a*_*p*_ are obtained via an optimisation function, but an analytic solution based on Bentler (1968) is also possible. The variance of the measurement error of each indicator needed for this optimization process was estimated by using the formulas presented in Section 2.2. The second method, further referred to as **WMW–mean**, also applies the WMW test on an aggregated indicator, but here the aggregation is simplified to the mean of the standardized indicators. This comparison enables us to study the influence of estimating weights according to the quality of the individual indicators. The third method uses the maximally reliable composite as input for a Welch *t*-test while the fourth method has the unweighted mean as input variable for a Welch *t*-test. These two methods are accordingly further referred to as ***t*****-test–max rel** and ***t*****-test–mean** and form a parametric alternative for the first and second method. The fifth and sixth method are based on SEM with equal group loadings and intercepts and are currently the most common methods to compare a latent variable between two groups. We include both SEM with and without correction for non-normal data, further referred as **SEM** and **SEM–correction**. The correction for non-normal data refers to the use of robust Satorra-Bentler standard errors (Satorra and Bentler, 1994). **SEM** and **SEM–correction** can also be seen as a parametric counterpart of the proposed WMW extension, but unlike the Welch *t*-test, SEM does take into account the measurement error of the indicators. The SEMs are implemented by using lavaan (Rosseel, 2012).

For sample size *m* = *n* = 15, *p*-values for the methods based on SEM and the Welch *t*-test were obtained by using a permutation null model, to ensure a fair comparison between the six different methods. For larger samples, inference was conducted by relying on the asymptotic distribution of the respective test statistics.

The R code to recreate the simulation study is available in the Supplementary Material.

### 3.1. Results

Because the Type I error rate is correctly controlled for almost all methods in almost all settings, we do not display these tables in the main text but provide them in the Supplementary Material (i.e., Tables 1–4 in Supplementary Data Sheet 2). For 3 cases, it can be noted that the methods relying on SEM are too liberal, i.e. the empirical Type I error rate is 6.9%. On the other hand, one can observe that the methods relying on the WMW test are too conservative in 4 cases for setting 2 with an empirical Type I error rate of 3.0 or 3.2%. These 7 deviations are indicated in bold in the corresponding tables in the Supplementary Material. It is noteworthy that even under model misspecification (i.e. setting 2 and 3) the Type I error rate is controlled for by all six methods. Hence, the assumption of distributionally homogeneous errors as postulated in Equation (5) is in practice less stringent. The results with respect to the empirical power are summarized in Tables 1–4.

**Table 1:** Empirical power from the simulation study with the indicators having an overall reliability of 80% and a linear relationship with the latent variable.

	**Linear**					

	**WMW–max rel**	**WMW–mean**	***t*** **-test–max rel**	***t*** **-test–mean**	**SEM**	**SEM–corrected**
N (0,1)						
***m* = *n* = 15**						
Setting 1	29.7	31.0	**33.2**	32.9	32.4	32.1
Setting 2	28.4	29.2	30.7	**31.3**	30.0	30.3
Setting 3	29.5	29.3	31.8	**32.3**	32.0	31.9
Setting 4	25.8	26.2	28.5	27.4	**29.4**	28.6
***m* = *n* = 50**						
Setting 1	78.7	79.1	81.7	81.4	**83.2**	83.1
Setting 2	79.5	78.8	81.0	80.4	82.0	**82.1**
Setting 3	79.2	79.9	81.2	81.8	**82.2**	82.0
Setting 4	74.8	72.3	77.6	74.4	**80.0**	**80.0**
***m* = *n* = 100**						
Setting 1	98.2	98.1	98.5	98.6	**98.7**	**98.7**
Setting 2	97.7	97.7	98.2	**98.3**	**98.3**	**98.3**
Setting 3	97.6	97.5	98.1	**98.2**	**98.2**	**98.2**
Setting 4	97.4	95.1	98.1	95.8	98.1	**98.2**
*t*_5_						
***m* = *n* = 15**						
Setting 1	23.5	23.9	24.2	24.1	**24.4**	23.9
Setting 2	24.0	**24.1**	23.1	22.6	22.0	22.2
Setting 3	23.7	**23.8**	22.4	23.1	22.5	23.3
Setting 4	22.1	22.4	22.2	22.4	22.4	**23.5**
***m* = *n* = 50**						
Setting 1	65.8	**66.1**	60.8	60.9	61.8	61.4
Setting 2	**68.7**	**68.7**	62.2	62.2	63.7	63.4
Setting 3	67.3	**67.6**	61.1	61.4	62.7	62.2
Setting 4	**63.9**	58.4	57.6	53.7	60.2	60.0
***m* = *n* = 100**						
Setting 1	92.3	**92.4**	87.6	87.6	88.0	88.1
Setting 2	**91.7**	**91.7**	87.0	87.4	87.9	87.8
Setting 3	93.2	**93.4**	88.9	88.7	89.4	89.5
Setting 4	**90.6**	86.8	85.7	83.4	87.4	87.4
	**Linear**					
	**WMW–max rel**	**WMW–mean**	***t*** **-test–max rel**	***t*** **-test–mean**	**SEM**	**SEM–corrected**
*Laplace* (0, 1.25)						
***m* = *n* = 15**						
Setting 1	21.5	**22.3**	19.6	20.4	19.3	19.0
Setting 2	**21.9**	21.4	21.0	21.1	19.8	21.0
Setting 3	22.0	**22.5**	20.6	20.3	20.1	20.3
Setting 4	17.3	**19.8**	16.5	18.8	19.4	19.3
***m* = *n* = 50**						
Setting 1	61.8	**62.0**	53.2	53.2	55.0	54.6
Setting 2	62.2	**63.2**	52.7	53.3	54.5	53.8
Setting 3	64.6	**66.0**	52.9	52.8	54.7	54.2
Setting 4	**58.9**	56.9	51.7	49.8	56.0	56.1
***m* = *n* = 100**						
Setting 1	**92.3**	92.2	83.2	82.9	83.5	83.4
Setting 2	**88.1**	87.9	77.7	77.5	78.2	78.1
Setting 3	**91.0**	90.7	82.6	82.9	82.9	83.0
Setting 4	**85.5**	80.4	77.7	74.2	79.3	79.3
Exp						
***m* = *n* = 15**						
Setting 1	22.2	**23.1**	18.1	18.2	18.7	18.3
Setting 2	19.7	**19.8**	17.0	17.5	17.2	17.5
Setting 3	19.4	**20.2**	16.9	17.6	16.9	17.0
Setting 4	15.7	**16.0**	13.4	14.9	14.8	14.9
*m* = *n* = 50						
Setting 1	61.9	**62.0**	40.8	41.8	42.6	42.6
Setting 2	56.2	**57.6**	38.0	38.2	39.9	39.3
Setting 3	63.0	**63.5**	42.6	42.6	44.9	44.8
Setting 4	**50.1**	45.0	36.9	36.7	40.6	40.4
***m* = *n* = 100**						
Setting 1	86.7	**87.2**	67.2	67.5	67.7	67.8
Setting 2	85.4	**85.6**	65.5	66.0	66.6	66.8
Setting 3	88.2	**88.4**	66.4	66.3	67.3	67.6
Setting 4	**83.5**	76.1	65.2	61.1	66.9	67.7

*The highest power per setting is indicated in bold*.

**Table 2:** Empirical power from the simulation study with the indicators having an overall reliability of 60% and a linear relationship with the latent variable.

	**Linear**					

	**WMW–max rel**	**WMW–mean**	***t*** **-test–max rel**	***t*** **-test–mean**	**SEM**	**SEM–corrected**
N (0,1)						
***m* = *n* = 15**						
Setting 1	26.4	27.7	28.8	**29.4**	28.5	28.9
Setting 2	23.9	25.4	26.0	**26.9**	25.4	24.6
Setting 3	26.4	27.4	28.5	**29.9**	28.9	28.3
Setting 4	21.1	24.1	23.5	**25.3**	24.0	23.7
***m* = *n* = 50**						
Setting 1	74.6	74.2	76.4	76.4	77.9	**78.1**
Setting 2	73.8	74.2	75.7	75.8	**77.0**	76.7
Setting 3	75.4	74.7	76.7	**77.8**	**77.8**	77.6
Setting 4	67.8	68.0	71.1	70.0	**74.5**	74.2
***m* = *n* = 100**						
Setting 1	96.7	97.0	97.5	97.6	**97.7**	**97.7**
Setting 2	95.9	96.2	**97.7**	**97.7**	**97.7**	**97.7**
Setting 3	95.8	95.7	96.3	96.3	**97.6**	**97.6**
Setting 4	94.4	92.3	95.1	94.0	95.6	**95.6**
*t*_5_						
***m* = *n* = 15**						
Setting 1	19.9	20.8	21.2	**21.8**	20.2	19.4
Setting 2	18.3	**19.6**	18.3	19.4	18.2	18.4
Setting 3	20.1	**20.8**	20.1	20.7	20.3	20.7
Setting 4	19.0	19.9	19.8	**21.4**	18.0	18.3
***m* = *n* = 50**						
Setting 1	56.4	**57.8**	54.2	54.8	55.4	55.4
Setting 2	61.6	**62.2**	56.7	57.0	58.2	58.1
Setting 3	**60.6**	**60.6**	57.4	58.1	59.0	58.2
Setting 4	**52.3**	51.7	49.1	48.4	51.2	51.5
***m* = *n* = 100**						
Setting 1	**87.7**	**87.7**	83.5	83.8	84.1	83.7
Setting 2	85.3	**86.3**	82.9	83.2	83.2	83.2
Setting 3	**89.1**	88.9	84.9	84.8	84.8	85.0
Setting 4	**83.7**	82.0	80.3	79.1	81.2	81.1
	**Linear**					
	**WMW–max rel**	**WMW–mean**	***t*** **-test–max rel**	***t*** **-test–mean**	**SEM**	**SEM–corrected**
*Laplace* (0, 1.25)						
***m* = *n* = 15**						
Setting 1	18.3	**19.0**	17.7	18.2	17.7	17.2
Setting 2	18.3	18.5	18.5	**19.0**	17.5	17.2
Setting 3	16.8	18.7	17.4	**18.9**	17.9	17.0
Setting 4	14.9	**17.7**	14.5	16.3	17.4	16.8
***m* = *n* = 50**						
Setting 1	51.6	**51.8**	47.4	48.0	48.4	48.7
Setting 2	54.0	**54.1**	48.7	49.0	49.9	50.6
Setting 3	55.9	**56.0**	47.2	47.2	48.9	49.2
Setting 4	49.0	48.0	46.7	45.5	**49.7**	**49.7**
***m* = *n* = 100**						
Setting 1	**85.5**	**85.5**	78.1	78.1	79.0	79.1
Setting 2	80.1	**80.8**	72.7	72.7	73.0	73.4
Setting 3	85.5	**85.9**	78.7	79.3	78.8	79.1
Setting 4	85.5	**85.9**	78.7	79.3	78.8	79.1
Exp						
***m* = *n* = 15**						
Setting 1	16.1	**16.9**	14.6	16.4	15.7	14.6
Setting 2	15.5	**15.6**	13.9	15.4	14.5	14.2
Setting 3	14.9	**16.3**	14.3	14.5	14.3	13.8
Setting 4	12.9	**14.1**	12.0	13.9	12.1	12.2
***m* = *n* = 50**						
Setting 1	**48.8**	48.2	36.5	37.3	37.7	37.4
Setting 2	43.6	**44.7**	33.3	33.4	34.7	34.8
Setting 3	48.8	**50.1**	38.1	38.6	40.0	39.8
Setting 4	**37.6**	37.3	31.3	32.5	33.2	33.3
***m* = *n* = 100**						
Setting 1	**75.2**	75.0	61.6	62.0	62.2	62.6
Setting 2	74.3	**75.2**	60.2	61.2	61.1	61.0
Setting 3	77.4	**78.8**	60.8	61.5	61.8	62.0
Setting 4	**71.0**	66.7	58.2	54.2	59.6	59.5

*The highest power per setting is indicated in bold*.

**Table 3:** Empirical power from the simulation study with the indicators having an overall reliability of 80% and a non-linear relationship with the latent variable.

	**Non-linear**					

	**WMW–max rel**	**WMW–mean**	***t*** **-test–max rel**	***t*** **-test–mean**	**SEM**	**SEM–corrected**
N (0,1)						
***m* = *n* = 15**						
Setting 1	29.7	30.3	**32.2**	31.4	31.8	31.0
Setting 2	28.1	29.0	29.3	**30.3**	28.7	28.6
Setting 3	28.1	28.4	30.6	**31.4**	30.8	29.8
Setting 4	23.7	25.5	25.7	26.2	**29.1**	27.0
***m* = *n* = 50**						
Setting 1	78.0	78.8	79.0	79.8	**80.7**	80.6
Setting 2	77.7	77.7	79.4	79.4	80.6	**81.2**
Setting 3	78.9	78.5	79.8	80.6	81.2	**81.3**
Setting 4	73.5	70.9	76.1	73.6	78.8	**79.1**
***m* = *n* = 100**						
Setting 1	97.6	97.7	98.2	98.2	**98.3**	98.2
Setting 2	97.6	97.5	97.9	97.9	**98.0**	**98.0**
Setting 3	97.3	96.9	**98.0**	97.8	**98.0**	**98.0**
Setting 4	96.3	94.8	97.1	95.4	**97.6**	**97.6**
*t*_5_						
***m* = *n* = 15**						
Setting 1	23.3	23.1	23.6	24.0	**24.1**	23.2
Setting 2	23.0	23.4	23.4	**24.1**	22.8	23.2
Setting 3	23.5	23.9	23.8	**24.2**	24.0	23.9
Setting 4	22.2	22.7	21.8	**23.3**	22.4	22.7
***m* = *n* = 50**						
Setting 1	65.4	**65.8**	60.8	61.1	62.2	62.1
Setting 2	68.5	**69.2**	63.6	63.3	64.8	64.9
Setting 3	66.5	**67.1**	62.9	62.9	64.5	65.1
Setting 4	**62.9**	57.5	58.5	55.2	61.4	61.1
***m* = *n* = 100**						
Setting 1	**92.2**	92.1	88.9	88.7	89.3	89.5
Setting 2	**91.6**	91.3	88.8	88.7	89.3	89.5
Setting 3	93.0	**93.2**	90.6	90.9	90.8	90.9
Setting 4	**90.4**	86.7	87.5	84.6	88.9	88.8
	**Non-linear**					
	**WMW–max rel**	**WMW–mean**	***t*** **-test–max rel**	***t*** **-test–mean**	**SEM**	**SEM–corrected**
*Laplace* (0, 1.25)						
***m* = *n* = 15**						
Setting 1	21.1	**22.3**	19.5	20.2	20.2	19.7
Setting 2	**22.9**	22.4	22.2	21.8	20.9	21.8
Setting 3	22.5	**22.7**	21.2	20.5	20.4	21.4
Setting 4	19.3	20.2	18.0	19.9	**21.0**	20.2
***m* = *n* = 50**						
Setting 1	61.6	**62.9**	53.9	55.1	55.9	55.4
Setting 2	62.1	**63.5**	55.4	55.8	56.3	56.8
Setting 3	64.4	**65.4**	55.6	55.4	57.4	57.9
Setting 4	**59.8**	56.2	54.4	50.1	57.0	56.9
***m* = *n* = 100**						
Setting 1	92.0	**92.6**	85.6	85.5	86.1	85.8
Setting 2	88.3	**89.0**	80.6	80.2	81.2	81.2
Setting 3	91.1	**91.7**	85.2	85.7	86.1	86.1
Setting 4	**86.1**	81.9	80.7	75.9	81.9	81.8
Exp						
***m* = *n* = 15**						
Setting 1	25.0	**25.9**	23.6	23.3	23.7	24.2
Setting 2	**23.1**	22.5	21.2	21.5	22.1	23.2
Setting 3	**22.4**	22.2	20.9	21.2	21.4	21.6
Setting 4	**19.4**	18.8	18.7	18.7	18.2	18.5
***m* = *n* = 50**						
Setting 1	**67.9**	**67.9**	58.3	57.1	60.8	59.7
Setting 2	63.7	**64.6**	54.5	54.0	56.8	56.9
Setting 3	69.6	**70.4**	60.6	59.1	62.3	61.7
Setting 4	**58.7**	55.9	49.7	49.0	54.8	54.6
***m* = *n* = 100**						
Setting 1	**91.8**	91.7	84.7	84.1	85.4	85.7
Setting 2	**89.5**	**89.5**	82.6	82.5	83.2	84.1
Setting 3	**92.8**	92.7	86.8	85.5	87.1	87.0
Setting 4	**90.5**	85.3	81.6	79.4	84.6	84.5

*The highest power per setting is indicated in bold*.

**Table 4:** Empirical power from the simulation study with the indicators having an overall reliability of 60% and a non-linear relationship with the latent variable.

	**Non-linear**					

	**WMW–max rel**	**WMW–mean**	***t*** **-test–max rel**	***t*** **-test–mean**	**SEM**	**SEM–corrected**
N (0,1)						
***m* = *n* = 15**						
Setting 1	24.6	25.6	27.7	**28.3**	27.5	28.0
Setting 2	22.6	24.6	25.1	**26.7**	24.6	24.2
Setting 3	24.0	26.3	26.8	**28.3**	26.6	25.9
Setting 4	21.2	21.6	23.1	**23.2**	**23.3**	**23.2**
***m* = *n* = 50**						
Setting 1	71.6	72.4	73.2	74.0	75.2	**75.3**
Setting 2	71.6	71.8	74.3	74.0	75.4	**75.5**
Setting 3	73.4	73.3	74.7	75.4	**76.2**	76.1
Setting 4	65.8	64.9	68.9	66.8	72.2	**72.3**
***m* = *n* = 100**						
Setting 1	95.4	95.8	96.5	**96.6**	**96.9**	96.8
Setting 2	94.9	95.0	96.1	96.0	96.1	**96.2**
Setting 3	94.9	94.6	95.5	95.6	95.7	**95.8**
Setting 4	92.6	91.5	94.0	92.2	94.6	**94.7**
*t*_5_						
***m* = *n* = 15**						
Setting 1	19.2	19.2	20.4	**21.2**	19.0	18.8
Setting 2	18.6	19.1	18.2	19.0	18.6	**19.3**
Setting 3	20.3	20.5	**21.1**	**21.1**	20.3	20.8
Setting 4	19.5	20.2	19.7	**20.6**	17.8	17.7
***m* = *n* = 50**						
Setting 1	56.6	56.6	55.9	56.1	56.6	**56.9**
Setting 2	60.0	**61.3**	57.1	57.3	57.7	57.8
Setting 3	**60.6**	60.2	57.8	58.3	58.9	59.2
Setting 4	51.9	50.8	51.0	49.2	52.0	**52.9**
***m* = *n* = 100**						
Setting 1	87.4	**87.7**	84.2	84.8	84.6	84.4
Setting 2	85.2	**85.9**	83.6	84.4	84.0	84.4
Setting 3	88.4	**88.9**	86.3	86.8	86.8	87.2
Setting 4	**84.2**	81.9	81.4	80.0	81.9	82.1
	**Non-linear**					
	**WMW–max rel**	**WMW–mean**	***t*** **-test–max rel**	***t*** **-test–mean**	**SEM**	**SEM–corrected**
*Laplace* (0, 1.25)						
***m* = *n* = 15**						
Setting 1	16.8	**19.0**	16.5	17.7	16.9	16.8
Setting 2	18.2	**19.2**	18.5	19.0	17.6	17.2
Setting 3	17.6	18.5	18.6	**19.3**	18.3	17.4
Setting 4	15.6	**17.4**	15.8	17.3	17.2	**17.4**
***m* = *n* = 50**						
Setting 1	50.9	**52.2**	48.4	49.3	49.4	49.3
Setting 2	52.8	**54.9**	49.8	51.1	51.2	51.8
Setting 3	56.0	**56.5**	49.0	49.3	51.2	51.8
Setting 4	49.5	49.6	46.8	46.5	**50.0**	49.5
***m* = *n* = 100**						
Setting 1	85.1	**85.8**	80.6	80.2	81.0	81.1
Setting 2	81.0	**81.6**	74.1	74.9	74.8	75.0
Setting 3	85.8	**86.1**	81.3	81.2	81.4	81.4
Setting 4	**78.1**	74.7	72.7	70.5	74.2	74.2
Exp						
***m* = *n* = 15**						
Setting 1	19.1	**21.4**	19.0	20.1	18.7	19.1
Setting 2	18.7	19.1	18.1	**19.4**	18.3	19.1
Setting 3	17.9	**19.0**	17.5	18.6	17.2	17.9
Setting 4	14.8	**16.8**	15.9	16.5	13.7	14.1
***m* = *n* = 50**						
Setting 1	58.0	**58.5**	51.2	52.3	53.3	53.1
Setting 2	52.9	**54.5**	46.9	49.3	48.6	49.2
Setting 3	58.3	**59.0**	51.7	53.2	54.1	53.1
Setting 4	46.2	**47.7**	43.0	44.0	44.5	44.4
***m* = *n* = 100**						
Setting 1	83.8	**84.6**	78.0	78.4	78.7	78.8
Setting 2	83.0	**84.1**	77.0	77.7	77.5	77.8
Setting 3	85.5	**86.5**	79.6	80.3	80.3	80.0
Setting 4	**80.0**	78.4	74.8	74.6	76.6	75.8

*The highest power per setting is indicated in bold*.

Regardless of the setting, sample size, reliability or type of function *h*_*p*_(·), the following trend can be observed with respect to the empirical power. When the latent variable is normally distributed, the six methods have similar power although the methods based on SEM and the *t*-test are slightly more powerful. As the distribution becomes more heavily tailed, the more superior the WMW methods become. This pattern is in accordance with the observations in the context of observed outcome variables, as mentioned by Van der Vaart (2000) and Hollander et al. (2013).

When one of the three indicators has an extremely low reliability, i.e. setting 4, a slightly different pattern can be seen. **SEM** has now a more pronounced superior power for the normal distribution. For the more heavily tailed distributions, the superiority of the WMW methods overall re-emerges. The methods **WMW–max rel** and **WMW–mean** differ in terms of the aggregated indicator that is used as input for the WMW method. The added value of the optimization process is demonstrated when looking at setting 4. The ability to fine-tune the weights in line with the reliability of each indicator separately and hence giving less influence to an indicator that has a bad quality, results in a higher empirical power for **WMW–max rel** in comparison with **WMW–mean**. However, it should be noted that when the reliability of indicators is equal to one another and/or the sample size is small (i.e. *m* = *n* = 15), a small reduction in empirical power for **WMW–max rel** in comparison with **WMW–mean** is observed. Hence, estimating the weights in order to optimize the estimated reliability of the aggregated indicator only has an added value when the data requires such adaptation.

Comparing the methods **SEM** and **SEM–correction**, the results with respect to the empirical power show no remarkable differences. Hence, the results suggest that the added value of robust Satorra-Bentler standard errors is limited in our simulation setup.

The lower the reliability of all three indicators, the lower the power, and this is true for all settings and all methods. To guard the readability of the tables, the point estimates and standard errors are not listed, but based on these results, the reason of the decrease in power is perhaps different between the WMW methods and SEM methods. For **SEM** and **SEM–correction**, it seems that there is barely an effect on the precision of the estimation but an increased empirical standard error is observed, hence influencing the power. Contrary, for **WMW–max rel** and **WMW–mean**, the empirical standard error remains relatively stable over the different levels of reliability, but the estimation is less accurate and hence influencing the power.

Overall, the simulation results suggest that the attractive properties of the original WMW method as discussed in the introduction are preserved in the context of latent variables. The extended WMW method is thus robust against outliers, relevant for skewed data and has a superior power in skewed distributions.

## 4. Illustration

We now reconsider the example of the introduction, where we want to examine the association between employment and depression in 455 patients with multiple sclerosis (MS).

**Figure 3:**
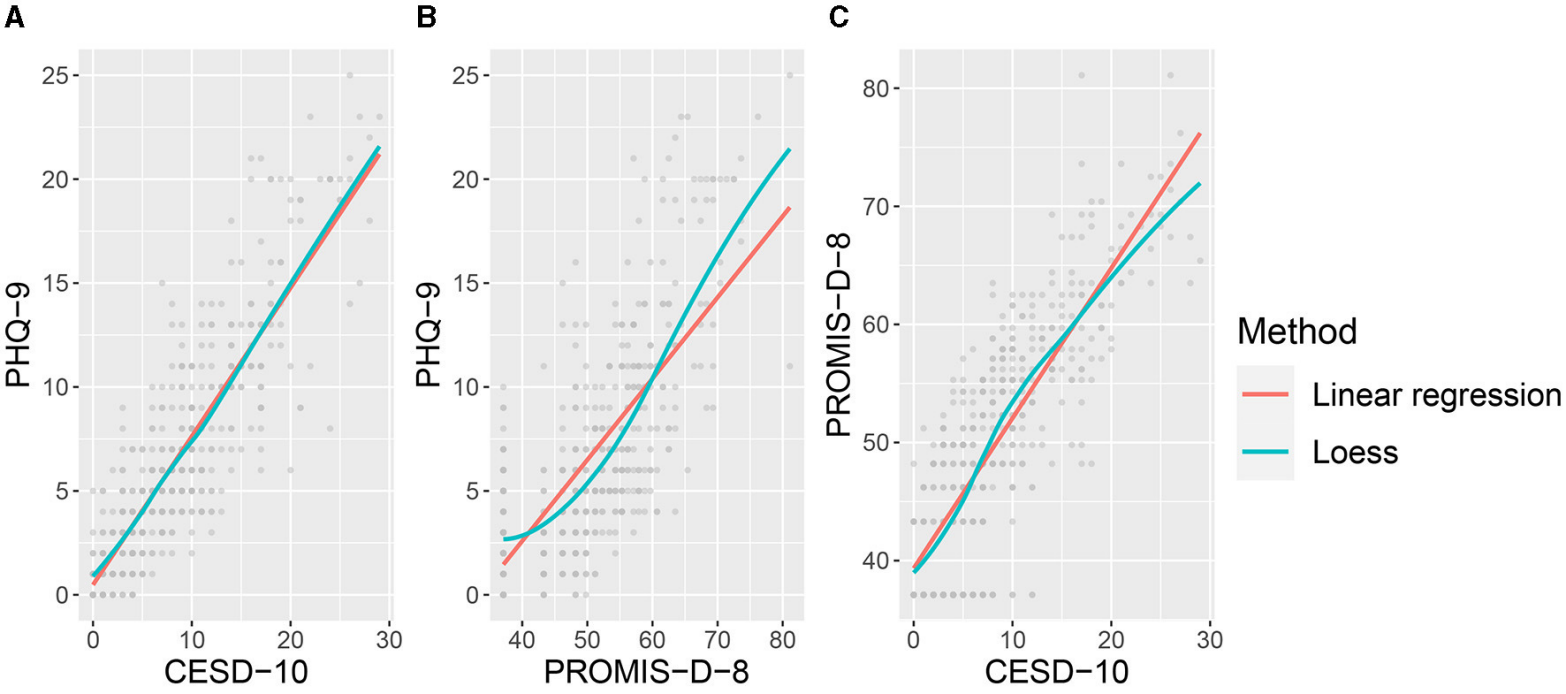
Figures **A–C** depict the pairwise scatterplots of the three questionnaires (i.e. PHQ-9, CESD-10 and PROMIS-D-8) that are the indicators for the latent variable depression. The scatterplots reveal deviations from linearity for the scatterplots depicting the PROMIS-D-8.

Figure 3 shows the pairwise scatterplots of the three depression questionnaires. The relation between PROMIS-D-8 and the other two indicators seems non-linear. This is in accordance with Amtmann et al. (2014), who argue that this questionnaire is subject to floor effects. Taking into account the characteristics of the data, i.e. the skewness depicted in Figure 1 and the observed non-linearity in Figure 3, the proposed extension of the WMW method is an appropriate analysis.

Visual inspection via Q–Q plots (see Figure 1 in Supplementary Data Sheet 2) shows that the assumption of location shift for the indicators is reasonably met. For the sake of completeness, the data are analyzed with the six estimation methods mentioned in the simulation study (Section 3): **WMW–max rel**, **WMW–mean**, ***t*****-test–max rel**, ***t*****-test– mean**, **SEM** and **SEM–correction**. In order to compare two groups by using our proposed WMW extension or via SEM, model comparison tests indicate that measurement invariance across the two groups is established. After performing a data-driven model evaluation, a residual covariance between CESD-10 and PROMIS-D-8 is added for both groups in order to further enhance the fit of the model when using SEM. Table 5 lists the *p*-values of all six methods and almost all methods lead to the conclusion that there is a significant difference in levels of depression between employed and unemployed people living with MS. Inference based on ***t*****-test–max rel** is only marginally significant at the 5% significance level.

**Table 5:** Effect sizes and inference based on the extended WMW methods, default and corrected SEM and the adapted *t*-tests when comparing the latent outcome variable depression between employed and unemployed patients with MS.

	**Probabilistic index**	***p*-value**
	**P** **(Depres_*unemployed*_>Depres_*employed*_)**	
WMW–max rel	56.48%	0.021
WMW–mean	56.92%	0.014
	**Mean difference**	***p*** **-value**
	**unemployed - employed**	
*t*-test–max rel	1.14	0.055
*t*-test–mean	0.19	0.034
SEM	1.444	0.0031
SEM–correction	1.444	0.0033

The interpretation of this difference does vary according to the used method. All parametric methods, i.e. the methods based on SEM and the *t*-test, give an estimation of the mean difference between the two groups where the employed patients are the reference group. Hence, a positive difference indicates that the unemployed patients have on average a higher score. Both **SEM** and **SEM–correction** give an estimated difference in latent means for depression of 1.444. Analyses based on the *t*-test with a weighted sum or mean of the standardized indicators result in a difference of 1.14 and 0.19, respectively. Given that these modified *t*-tests do not take a measurement model into account, in contrast with SEM, its effect size merely reflects a difference in an overall outcome that aims to measure depression. Nevertheless, both analyses indicate that the group of unemployed patients score on average higher for depression than the group of employed patients.

For the extended WMW methods, the interpretation of the effect sizes is slightly different. Here, the effect size represents the probability that an unemployed patient has a higher score for depression than an employed patient. These probabilities are 56.48 and 56.92% for respectively **WMW–max rel** and **WMW–mean**. Consequently, patients who are unemployed have a significantly higher probability (around 56%) to have higher depression scores than patients that do have a job. The results thus show that the conclusion in this specific context for all methods points in the same direction. The R code for all analysis and figures is made available in the Supplementary Material.

## 5. Discussion

In this paper, we proposed an extension of the Wilcoxon–Mann–Whitney test in the context of latent variables with a main focus on hypothesis testing. We introduced two strategies: one where the mean of the standardized indicators is used and one where a maximally reliable composite is created to form the input of the WMW test. The statistical properties of these proposed testing procedures were examined and compared with the performance of SEMs and two adapted *t*-tests in a simulation study. By comparing the two proposed strategies to each other in this simulation study, the costs and benefits of this maximization procedure were explored.

Even though SEM is omnipresent in the context of analyzing latent variables, we believe that a valuable alternative is provided in this paper. Concerning measurement model (1), the difference with the classical theory in SEM is the amount of knowledge that is required concerning the function *h*_*p*_(·). Typically, *h*_*p*_(·) is assumed to be linear and is estimated during the analysis. In our proposed methodology where the mean of the standardized indicators is used, we relax this requirement since we only assume monotonicity and we do not need to estimate *h*_*p*_(·) as this is not our main interest. Some flexibility has thus been introduced in comparison with the traditional FA and SEM. Additionally, the variances of the measurement error do not need to be estimated in this strategy. For the theoretical validation of our methodology, we do need to impose the assumption of equal distributions for the measurement error per indicator across two groups. However, the simulation results suggest that violations have no detrimental consequences with respect to both the empirical Type I error rate and the power.

When a maximally reliable composite is used, i.e. the second strategy of the extended WMW test, the variance of the measurement error needs to be estimated in order to obtain the weights for this aggregated indicator. In the proposed formulas for the estimation of the variance, linearity among the indicators was imposed. Also here, deviations from this linearity assumption did not lead to problems with respect to hypothesis testing in our simulation study as long as the function *h*_*p*_(·) is monotone. It may be clear that the use of a maximally reliable composite requires additional assumptions in comparison with the use of a simple mean, but based on our simulation study, these assumptions seem to be flexible in practice.

Using an aggregated indicator based on the data-driven maximization procedure entails an improvement compared with the use of an unweighted mean when the reliability of the indicators differs. These results confirm the theoretical expectation that fine-tuning the weights in line with the reliability of each indicator separately can result in an increase in empirical power. However, one should not needlessly use this maximization procedure as it can result in a small loss of power when e.g. the reliability of indicators is equal to one another and/or the sample size is small (i.e. *m* = *n* = 15).

Most interestingly for this paper is that the results show that the attractive properties of the original WMW method are transferred to the context of latent variables. The results confirm that the procedures based on the WMW test have superior power when the distribution is heavily tailed. This is a pattern that is also observed when simulating data in the context of observed outcome variables, as mentioned by Van der Vaart (2000) and Hollander et al. (2013).

A possible limitation for the user might be the effect size of the extended WMW test. Where SEM provides an estimate of the difference between two groups on the scale of the latent variable which can be standardized, the effect size of our proposed method is a probabilistic index. On the other hand, from a theoretical point of view, this latter effect size has attractive properties. A probabilistic index is scale invariant and robust to outliers.

A second limitation of this study is the sole focus on hypothesis testing. It is known that using the standard Wilcoxon–Mann–Whitney test in the context of measurement error leads to an underestimation of the true effect size (Coffin and Sukhatme, 1996, 1997; Faraggi, 2000; Schisterman et al., 2001; Tosteson et al., 2005; Fuller, 2009). In future research, adaptations to the proposed methodology can be studied to enhance the point estimation. Other possible directions for research are extensions for paired groups, multiple group comparison or comparing groups over time.

A third limitation of the suggested methodology is that it is inherently impossible to model the monotonic relation between the indicator and the latent variable, in contrast to SEM. The methodology presented in this paper extends the standard Wilcoxon–Mann–Whitney method and hence only uses the rank of the data. Closely related is the remark that the extended WMW test can only be applied after measurement invariance is determined by using SEM. The additional use of the extended WMW test is especially justified when the distribution is heavily tailed in order to profit from the attractive properties of the extended WMW test as discussed earlier.

To conclude, this paper validated the use of the WMW method in the context of latent variables by implementing some small adaptations, i.e. the creation of an aggregated indicator. The use of existing concepts not only facilitates the practical implementation for researchers and practitioners, the advantages of the original WMW method are also carried over into the new context. We believe that the combination of flexibility in the measurement model, the ability to allocate weights reflecting the reliability of indicators and the superiority in heavily tailed distributions results in a valuable methodology.

## Data Availability Statement

Requests to access data with respect to the simulation study should be directed to Heidelinde Dehaene, heidelinde.dehaene@ugent.be. Some previously collected existing data was used in this article and is not publicly available. Therefore, requests to access these data should be directed to the corresponding authors [i.e. Amtmann et al. (2014)].

## Author Contributions

HD, JDN, and YR: conceptualization and methodology of the presented idea. HD: implementation of the simulation studies, data analysis and writing of the manuscript. JDN and YR: review and editing of the manuscript and supervision. All authors approved the submitted version.

## Funding

This work was financially supported by a Special Research Fund (BOF) Starting Grant 01N00717 from Ghent University. The computational resources (Stevin Supercomputer Infrastructure) and services used in this work were provided by the VSC (Flemish Supercomputer Center), funded by Ghent University, FWO and the Flemish Government–department EWI.

## Conflict of Interest

The authors declare that the research was conducted in the absence of any commercial or financial relationships that could be construed as a potential conflict of interest.

## Publisher's Note

All claims expressed in this article are solely those of the authors and do not necessarily represent those of their affiliated organizations, or those of the publisher, the editors and the reviewers. Any product that may be evaluated in this article, or claim that may be made by its manufacturer, is not guaranteed or endorsed by the publisher.

## Supplementary Material

The R code to recreate the simulation study and to reproduce all analysis and figures of the case study is made available in the Supplementary Material.

## References

[B1] Amtmann D. Kim J. Chung H. Bamer A. M. Askew R. L. Wu S.. (2014). Comparing CESD-10, PROMIS-9, and PROMIS depression instruments in individuals with multiple sclerosis. Rehabil. Psychol. 59:220. 10.1037/a0035919 24661030 PMC4059037

[B2] Andresen E. M. Malmgren J. A. Carter W. B. Patrick D. L. (1994). Screening for depression in well older adults: evaluation of a short form of the CES-D. Am. J. Prev. Med. 10, 77–84. 10.1016/S0749-3797(18)30622-6 8037935

[B3] Bentler P. (1968). Alpha-maximized factor analysis (alphamax): Its relation to alpha and canonical factor analysis. Psychometrika 33, 335–345. 10.1007/BF02289328 5243965

[B4] Blair R. C. Higgins J. J. (1980). A comparison of the power of wilcoxon's rank-sum statistic to that of student's t statistic under various nonnormal distributions. J. Educ. Stat. 5, 309–335. 10.2307/1164905

[B5] Chou C.-P. Bentler P. M. Satorra A. (1991). Scaled test statistics and robust standard errors for non-normal data in covariance structure analysis: a monte carlo study. Br. J. Math. Stat. Psychol. 44, 347–357. 10.1111/j.2044-8317.1991.tb00966.x 1772802

[B6] Coffin M. Sukhatme S. (1996). A parametric approach to measurement errors in receiver operating characteristic studies, in Lifetime Data: Models in Reliability and Survival Analysis (Boston, MA: Springer), 71–75.

[B7] Coffin M. Sukhatme S. (1997). Receiver operating characteristic studies and measurement errors. Biometrics 53, 823–837. 10.2307/2533545 9333348

[B8] Cohen J. (1988). Statistical Power Analysis for the Behavioral Sciences, 2nd Edn. Hillsdale, NJ: Erlbaum.

[B9] De Neve J. Dehaene H. (2021). Semiparametric linear transformation models for indirectly observed outcomes. Stat. Med. 40, 2286–2303. 10.1002/sim.8903 33565108

[B10] De Schryver M. De Neve J. (2019). A tutorial on probabilistic index models: Regression models for the effect size P(Y1 < Y2). Psychol. Methods 24:403. 10.1037/met0000194 30265047

[B11] Faraggi D. (2000). The effect of random measurement error on receiver operating characteristic (roc) curves. Stat. Med. 19, 61–70. 10.1002/(SICI)1097-0258(20000115)19:1<61::AID-SIM297>3.0.CO;2-A 10623913

[B12] Fuller W. A. (2009). Measurement Error Models, Vol. 305. New York, NY: John Wiley & Sons.

[B13] Garcia-Marques L. Garcia-Marques T. Brauer M. (2014). Buy three but get only two: The smallest effect in a 2 ×2 anova is always uninterpretable. Psychon. Bull. Rev. 21, 1415–1430. 10.3758/s13423-014-0640-3 24841234

[B14] Hollander M. Wolfe D. A. Chicken E. (2013). Nonparametric Statistical Methods, Vol. 751. John Wiley & Sons.

[B15] Jackson D. L. (2001). Sample size and number of parameter estimates in maximum likelihood confirmatory factor analysis: a monte carlo investigation. Struct. Equ. Model. 8, 205–223. 10.1207/S15328007SEM0802_3

[B16] Kline R. B. (2015). Principles and Practice of Structural Equation Modeling. New York, NY: Guilford Publications.

[B17] Kroenke K. Spitzer R. L. (2002). The PHQ-9: a new depression diagnostic and severity measure. Psychiatr. Ann. 32, 509–515. 10.3928/0048-5713-20020901-06

[B18] Kroenke K. Spitzer R. L. Williams J. B. (2001). The PHQ-9: validity of a brief depression severity measure. J. Gen. Intern. Med. 16, 606–613. 10.1046/j.1525-1497.2001.016009606.x 11556941 PMC1495268

[B19] Lehmann E. L. (1951). Consistency and unbiasedness of certain nonparametric tests. Ann. Math. Stat. 22, 165–179. 10.1214/aoms/1177729639

[B20] Li H. (1997). A unifying expression for the maximal reliability of a linear composite. Psychometrika 62, 245–249. 10.1007/BF02295278

[B21] Mann H. B. Whitney D. R. (1947). On a test of whether one of two random variables is stochastically larger than the other. Ann. Math. Stat. 18, 50–60. 10.1214/aoms/1177730491

[B22] Nunnally J. C. Bernstein I. H. (1994). Psychometric Theory, 3rd Edn. New York, NY: McGraw-Hill.

[B23] Penev S. Raykov T. (2006). Maximal reliability and power in covariance structure models. Br. J. Math. Stat. Psychol. 59, 75–87. 10.1348/000711005X68183 16709280

[B24] Pilkonis P. A. Choi S. W. Reise S. P. Stover A. M. Riley W. T. Cella D. PROMIS Cooperative Group. (2011). Item banks for measuring emotional distress from the Patient-Reported Outcomes Measurement Information System (PROMIS®): depression, anxiety, and anger. Assessment 18, 263–283. 21697139 10.1177/1073191111411667 PMC3153635

[B25] R Core Team (2020). R: A Language and Environment for Statistical Computing. Vienna: R Foundation for Statistical Computing.

[B26] Rosseel Y. (2012). lavaan: an R package for structural equation modeling. J. Stat. Softw. 48, 1–36. 10.18637/jss.v048.i0225601849

[B27] Satorra A. Bentler P. M. (1994). Corrections to test statistics and standard errors in co-variance structure analysis, in Latent Variables Analysis: Applications for Developmental Research, eds A. von Eye and C. C. Clogg (Thousands Oaks: Sage), 399–419.

[B28] Schisterman E. F. Faraggi D. Reiser B. Trevisan M. (2001). Statistical inference for the area under the receiver operating characteristic curve in the presence of random measurement error. Am. J. Epidemiol. 154, 174–179. 10.1093/aje/154.2.174 11447052

[B29] Spitzer R. L. Kroenke K. Williams J. B. Group P. H. Q. P. C. S. (1999). Validation and utility of a self-report version of PRIME-MD: the PHQ primary care study. JAMA 282, 1737–1744. 10.1001/jama.282.18.1737 10568646

[B30] Thas O. (2010). Comparing Distributions. New York, NY: Springer.

[B31] Tosteson T. D. Buonaccorsi J. P. Demidenko E. Wells W. A. (2005). Measurement error and confidence intervals for roc curves. Biometr. J. 47, 409–416. 10.1002/bimj.200310159 16161800

[B32] Van der Vaart A. W. (2000). Asymptotic Statistics, Vol. 3. Cambridge: Cambridge University Press.

[B33] Wilcoxon F. (1945). Individual comparisons by ranking methods. Biometr. Bull. 1, 80–83. 10.2307/3001968

