# Supplementary figures and images for: A Wilcoxon–Mann–Whitney Test for Latent Variables
Source: Front Psychol. 2021 Nov 15;12:754898. doi: 10.3389/fpsyg.2021.754898 (PMC8634887; doi:10.3389/fpsyg.2021.754898)

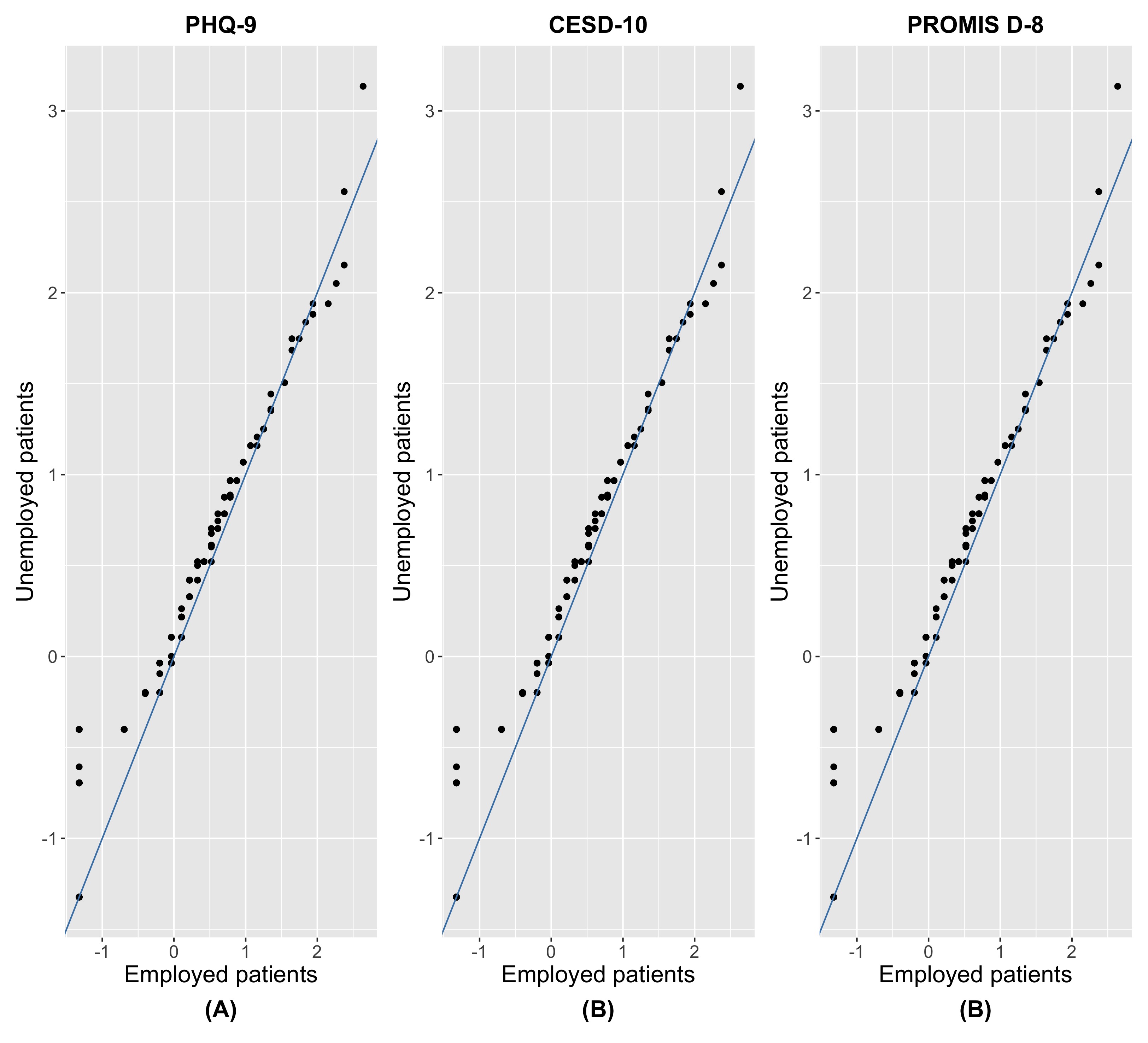

Supplement: Supplementary file 1 [file Data_Sheet_1.ZIP › R code/Manuscript - figures/Figure4.jpeg]

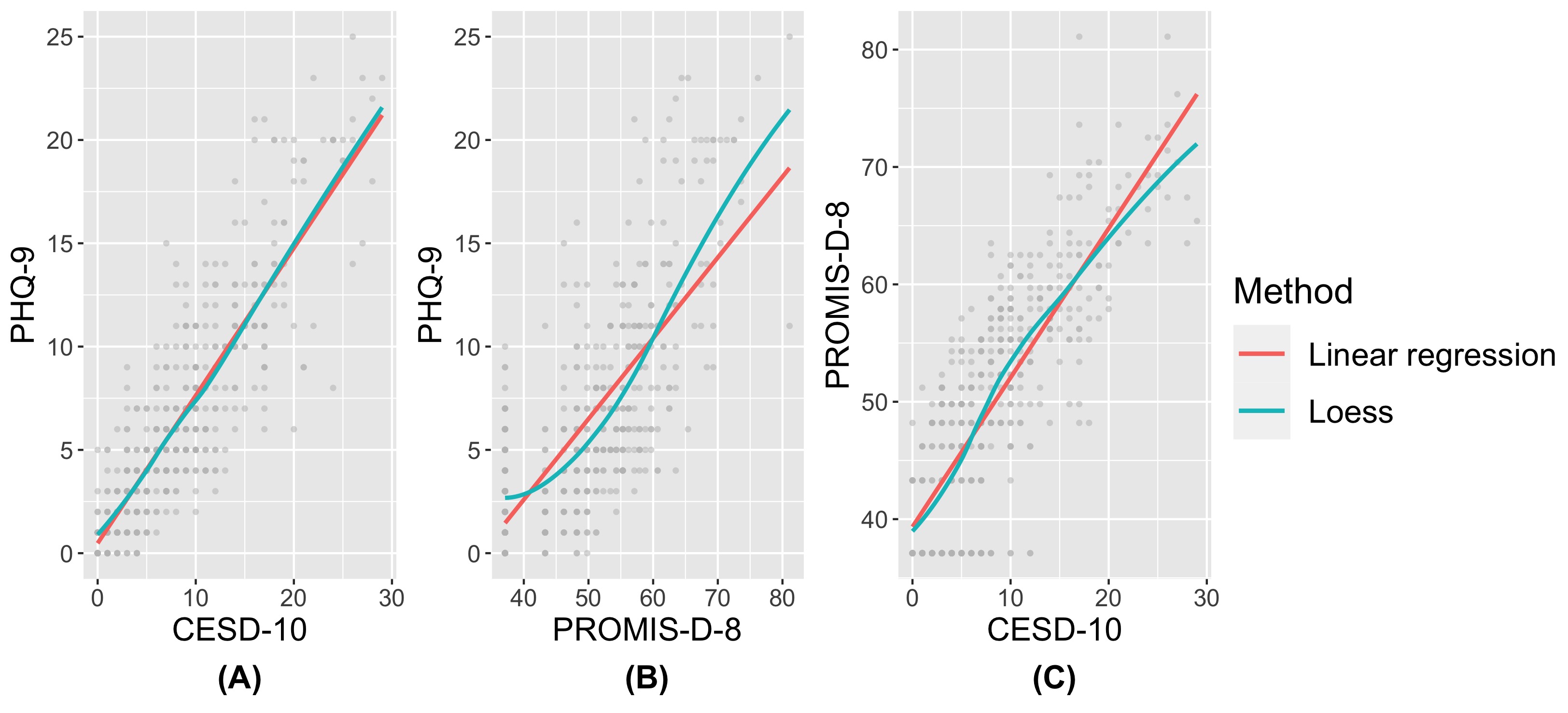

Supplement: Supplementary file 1 [file Data_Sheet_1.ZIP › R code/Manuscript - figures/Figure3.jpeg]

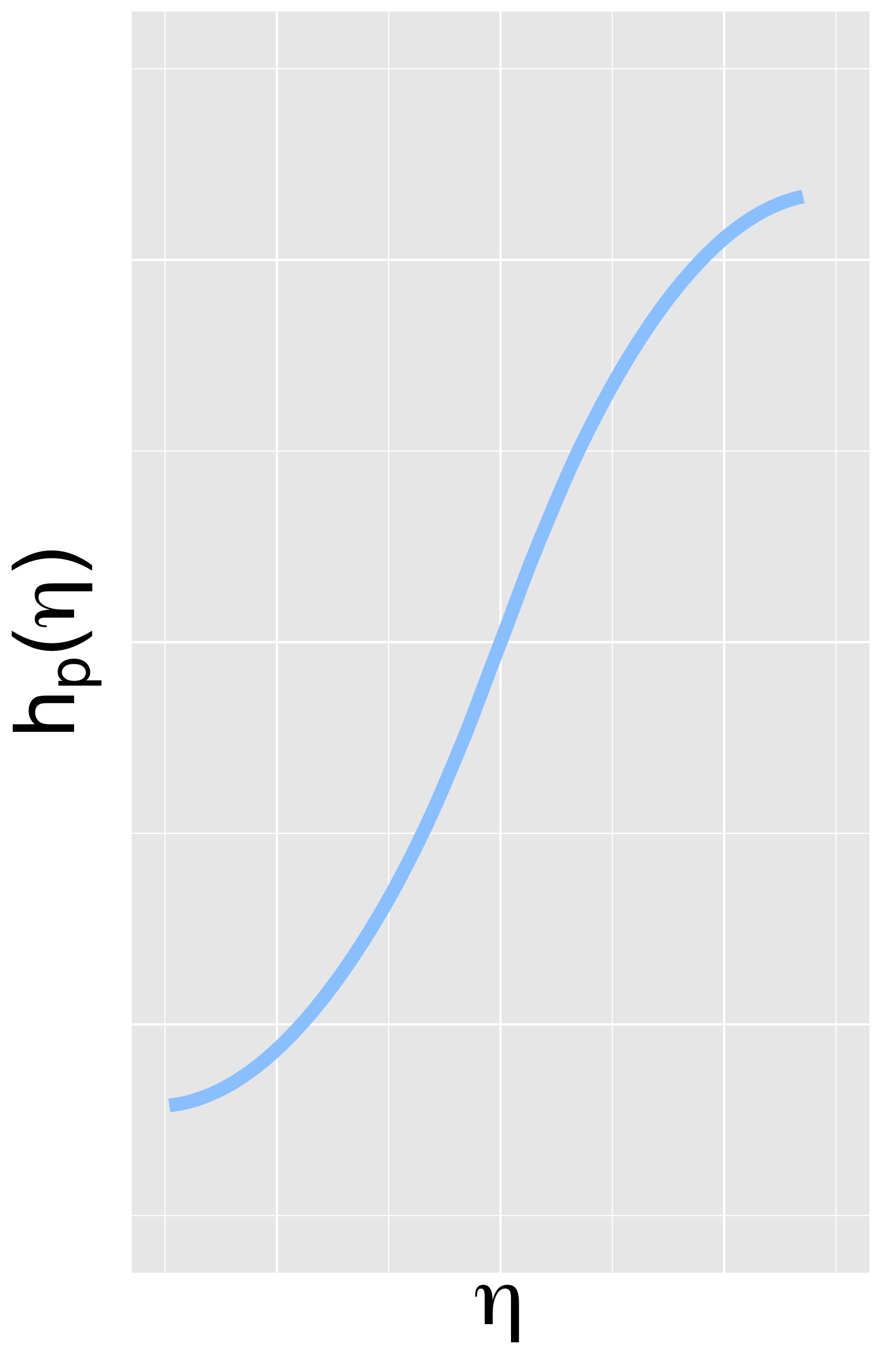

Supplement: Supplementary file 1 [file Data_Sheet_1.ZIP › R code/Manuscript - figures/Figure2.jpeg]

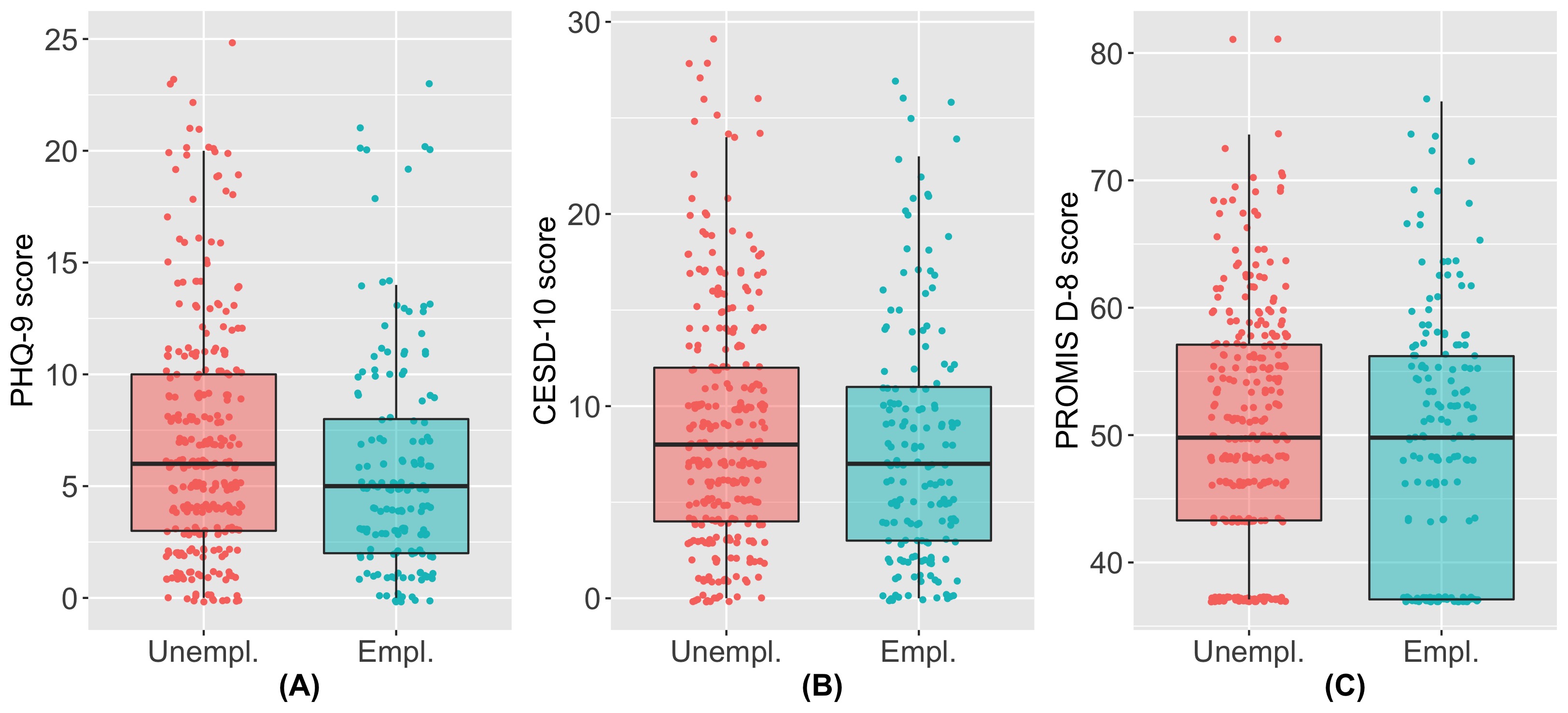

Supplement: Supplementary file 1 [file Data_Sheet_1.ZIP › R code/Manuscript - figures/Figure1.jpeg]

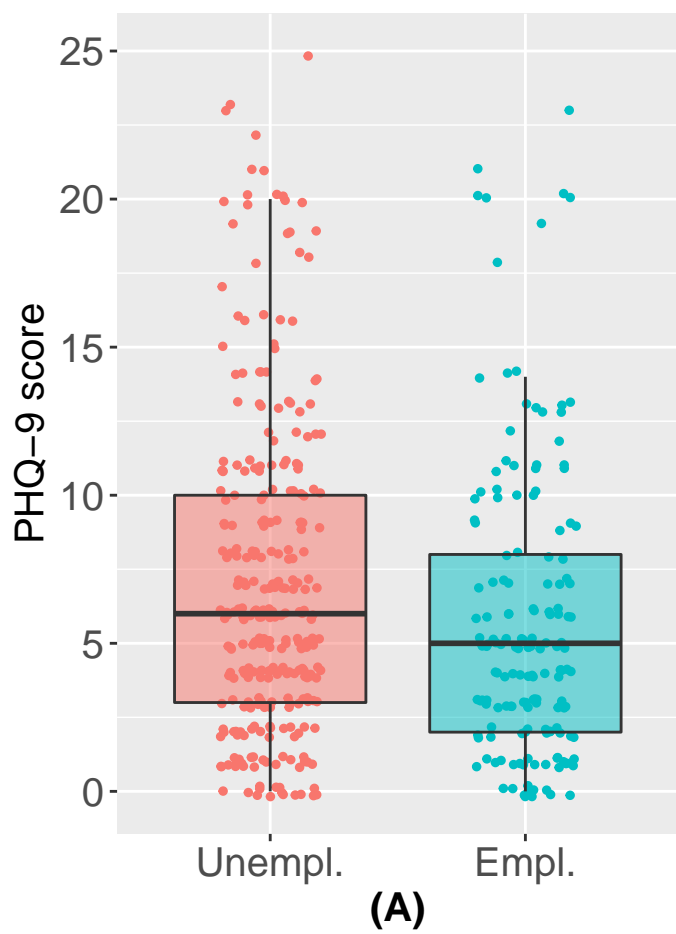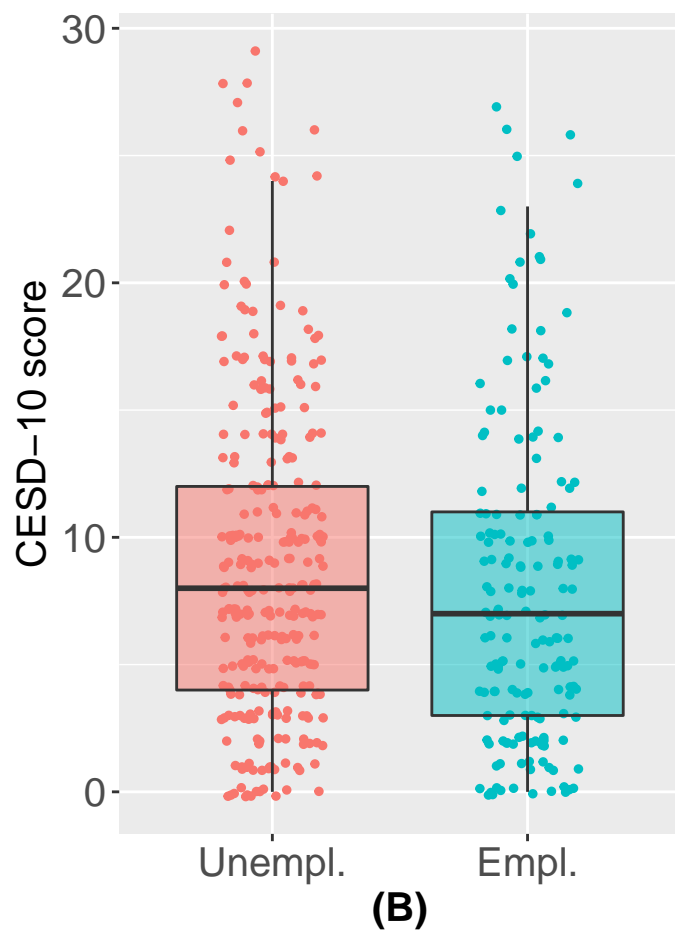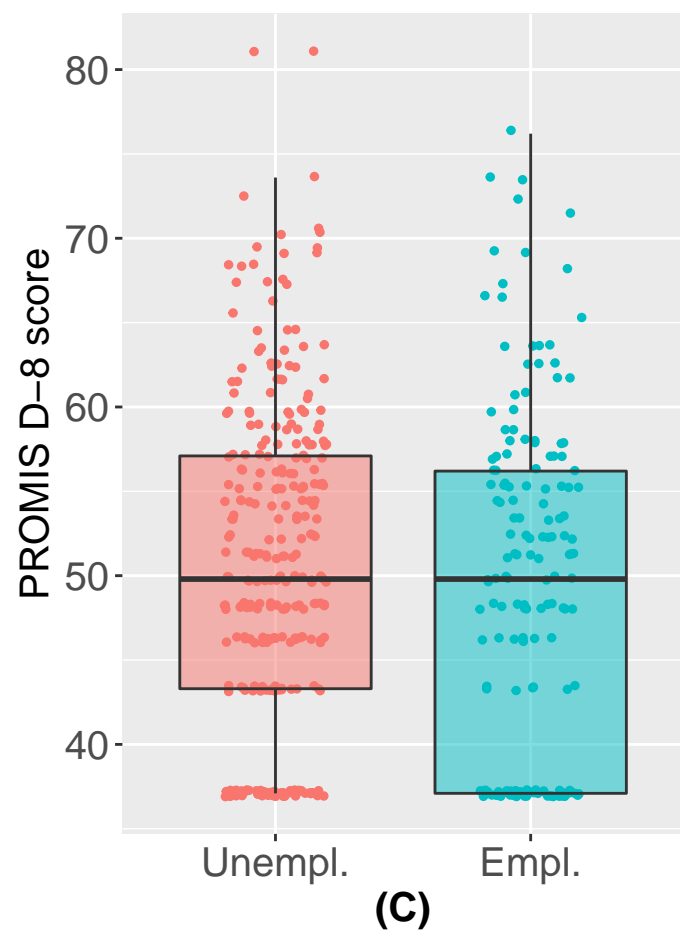

Supplement: Supplementary file 1 [file Data_Sheet_1.ZIP › R code/Manuscript - figures/Figure1.pdf]

$h_p(\eta)$

$\eta$

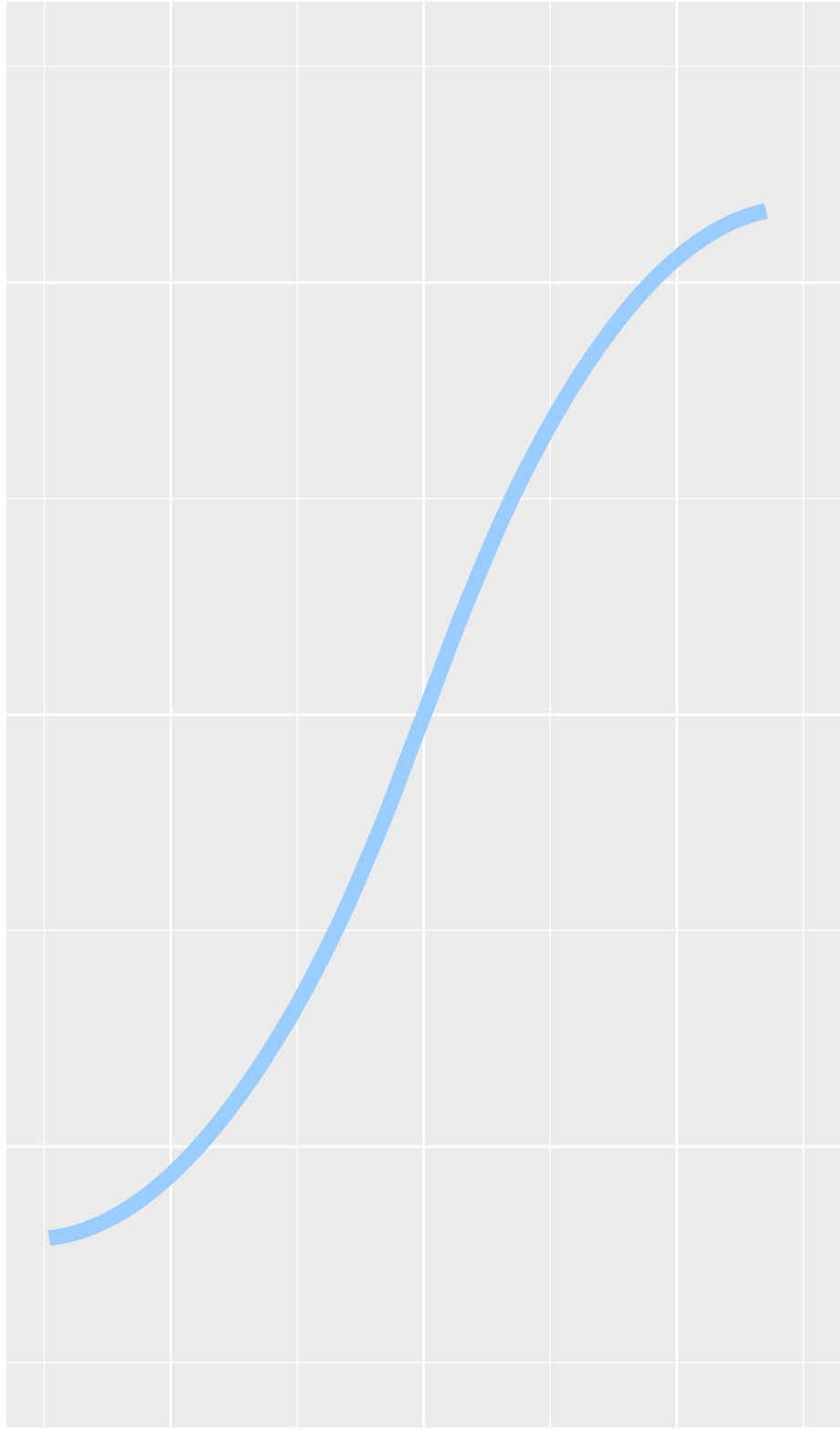

Supplement: Supplementary file 1 [file Data_Sheet_1.ZIP › R code/Manuscript - figures/Figure2.pdf]

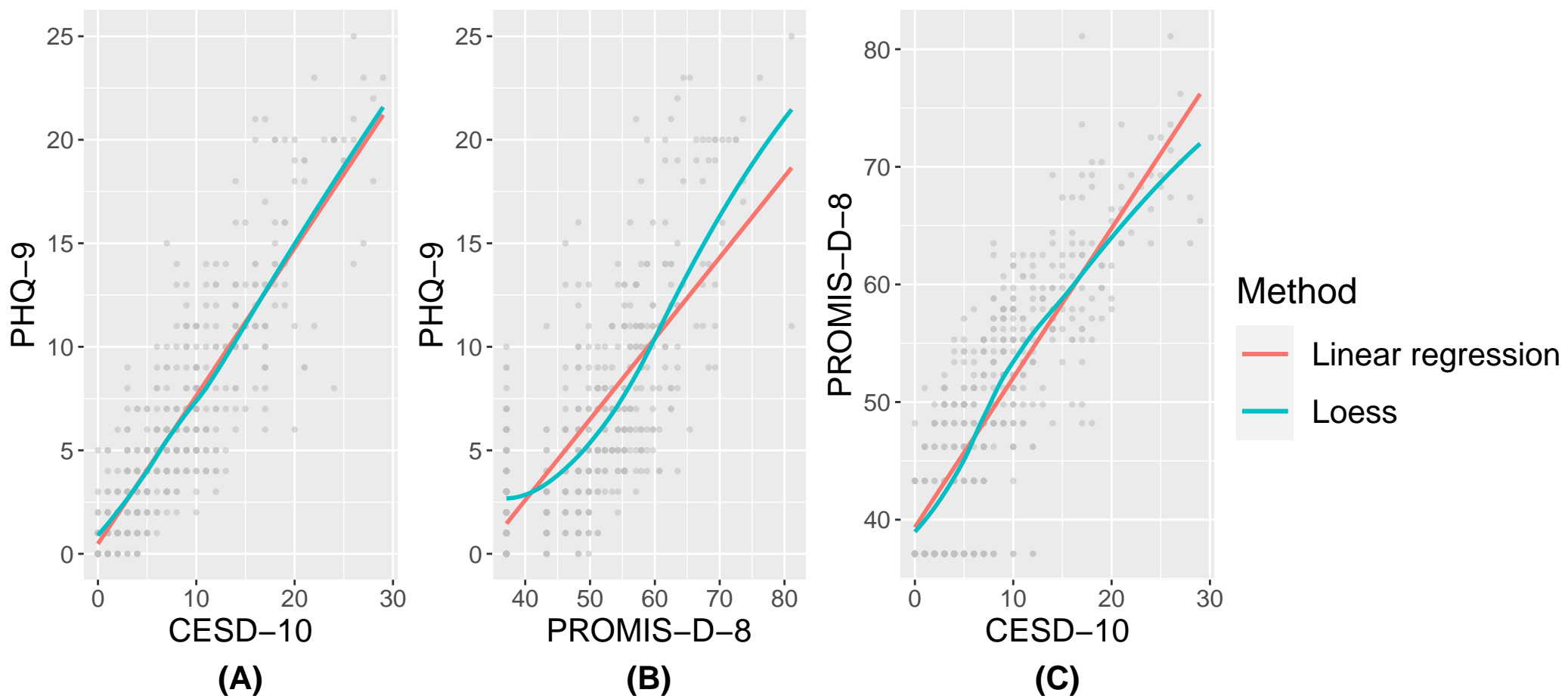

Supplement: Supplementary file 1 [file Data_Sheet_1.ZIP › R code/Manuscript - figures/Figure3.pdf]

**PHQ-9**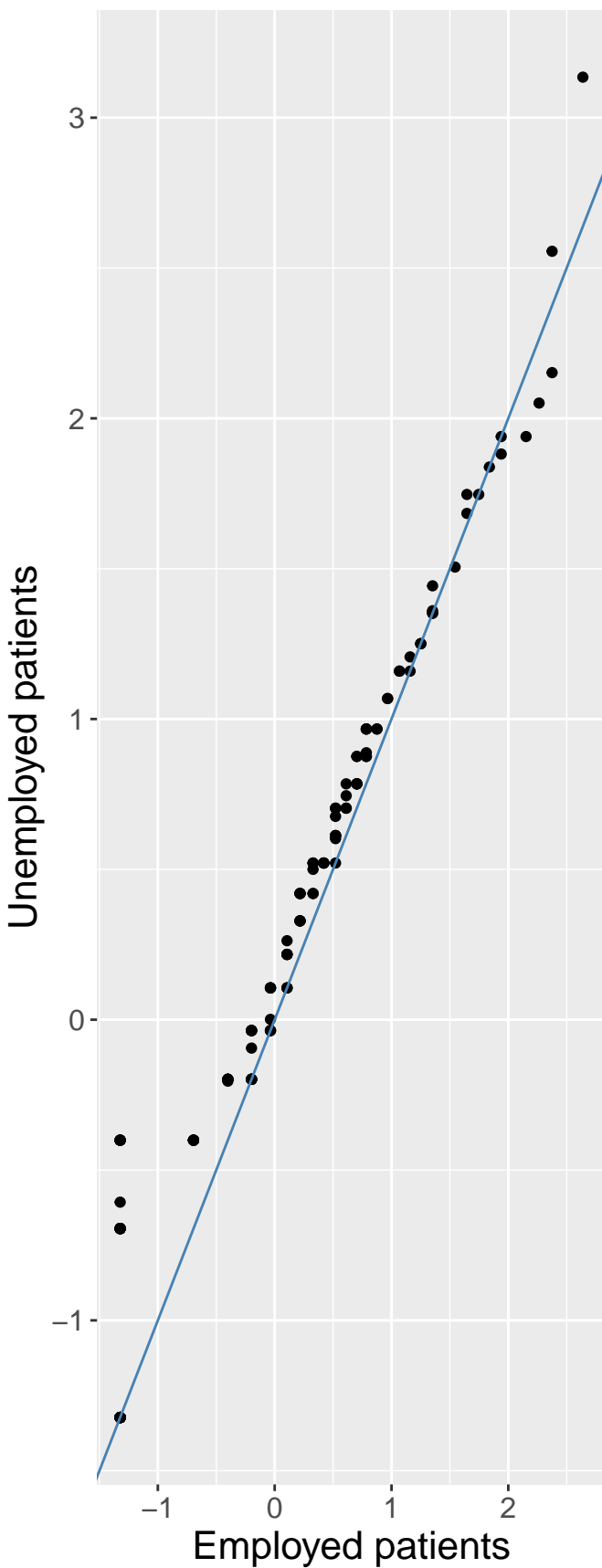**(A)****CESD-10**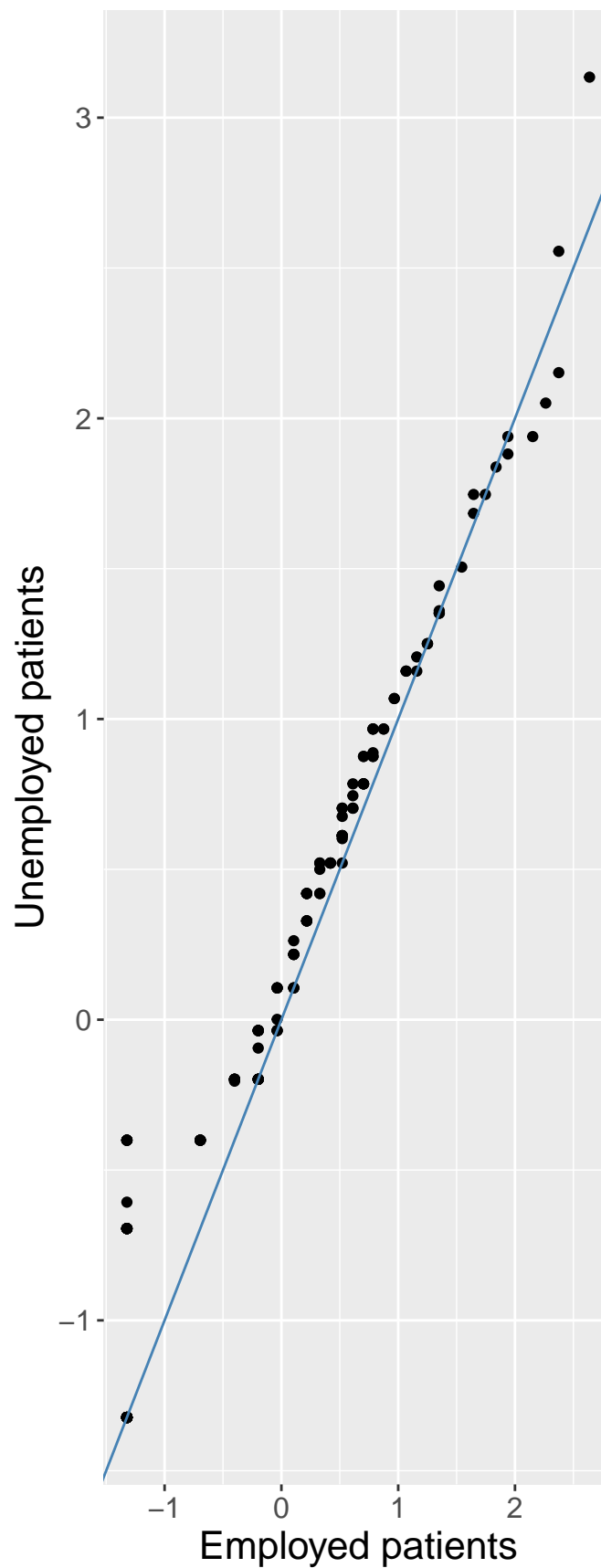**(B)****PROMIS D-8**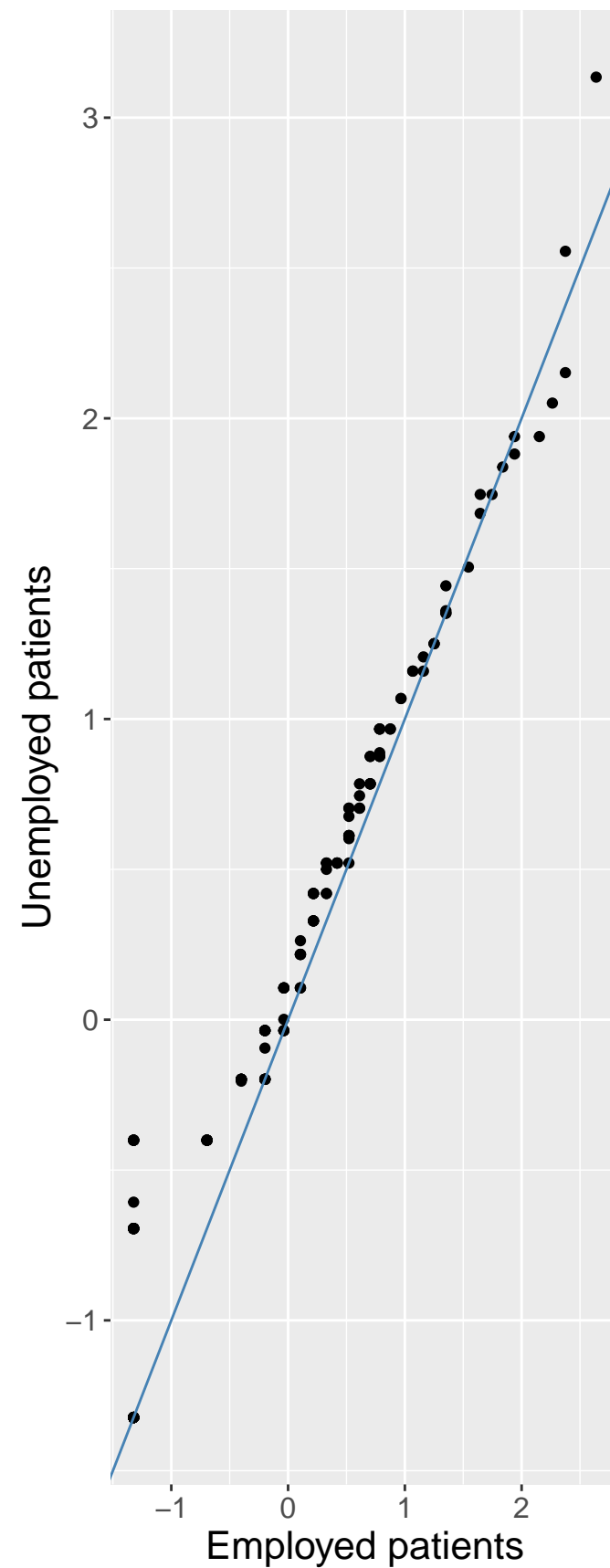**(B)**

Supplement: Supplementary file 1 [file Data_Sheet_1.ZIP › R code/Manuscript - figures/Figure4.pdf]
