# Supplementary material for: A Wilcoxon–Mann–Whitney Test for Latent Variables
Source: Front Psychol. 2021 Nov 15;12:754898. doi: 10.3389/fpsyg.2021.754898 (PMC8634887; doi:10.3389/fpsyg.2021.754898)
Supplement: Supplementary file 2 [file Data_Sheet_2.pdf]

Supplementary Material

1 RESULTS SIMULATION STUDY: TYPE I ERROR RATE

Table S1. Empirical Type I error rate from the simulation study with the indicators having an overall reliability of 80% and a linear relationship with the latent variable. Extreme deviations from 5% are indicated in bold.

	**Linear**					
	**WMW – max rel**	**WMW – mean**	***t* test – max rel**	***t* test – mean**	**SEM**	**SEM – corrected**
$\mathcal{N}(0,1)$						
$m = n = 15$						
Setting 1	4.8	5.0	5.2	5.5	5.2	4.9
Setting 2	4.4	4.4	4.3	4.7	4.8	4.5
Setting 3	4.7	4.6	5.0	5.5	5.3	5.1
Setting 4	4.7	5.1	5.9	6.3	6.2	6.2
$m = n = 50$						
Setting 1	4.8	4.8	5.3	4.8	6.1	5.8
Setting 2	5.3	5.1	6.4	6.0	6.6	6.5
Setting 3	4.3	3.8	4.8	4.6	5.1	5.1
Setting 4	5.6	5.2	6.0	5.3	6.8	**6.9**
$m = n = 100$						
Setting 1	3.9	3.9	3.8	3.8	3.8	3.7
Setting 2	6.2	6.1	5.6	5.3	5.6	5.6
Setting 3	5.5	5.5	6.1	6.0	6.3	6.3
Setting 4	4.6	6.0	4.2	5.7	5.1	5.0
t_5						
$m = n = 15$						
Setting 1	3.9	4.1	4.4	4.5	4.3	4.2
Setting 2	4.6	4.4	4.8	5.3	5.3	5.4
Setting 3	4.0	4.5	4.0	4.5	4.8	4.3
Setting 4	4.5	4.6	4.8	5.1	5.1	5.1
$m = n = 50$						
Setting 1	5.4	5.2	4.8	4.6	5.2	5.5
Setting 2	**3.2**	**3.2**	4.2	4.0	4.3	4.1
Setting 3	5.0	4.9	4.2	4.4	4.8	4.5
Setting 4	4.0	4.2	3.3	3.9	4.6	4.7
$m = n = 100$						
Setting 1	4.9	5.0	3.3	3.5	3.5	3.6
Setting 2	4.9	4.8	4.4	4.4	4.7	4.5
Setting 3	4.4	4.6	4.5	4.2	4.5	4.4
Setting 4	4.4	6.0	5.2	6.2	6.0	6.1

	**Linear**					
	**WMW – max rel**	**WMW – mean**	***t* test – max rel**	***t* test – mean**	**SEM**	**SEM – corrected**
$Laplace(0, 1.25)$						
$m = n = 15$						
Setting 1	4.8	5.2	5.8	5.8	5.4	5.4
Setting 2	3.8	3.7	4.4	3.9	4.8	4.4
Setting 3	4.7	4.9	5.2	5.3	5.4	5.9
Setting 4	4.1	4.1	4.7	4.5	5.1	5.4
$m = n = 50$						
Setting 1	5.0	5.3	5.6	5.6	6.0	6.1
Setting 2	4.7	5.5	4.6	4.7	5.2	5.4
Setting 3	4.6	4.7	4.6	5.1	5.3	5.5
Setting 4	5.2	4.4	5.2	5.0	6.3	5.9
$m = n = 100$						
Setting 1	5.1	5.5	3.9	3.8	4.1	4.2
Setting 2	5.0	5.2	5.5	5.1	5.8	5.8
Setting 3	5.1	5.4	5.3	5.3	5.5	5.7
Setting 4	5.0	4.7	3.7	4.2	4.5	4.5
Exp						
$m = n = 15$						
Setting 1	4.5	4.8	5.6	5.3	5.6	6.0
Setting 2	4.0	3.8	5.4	4.7	5.1	5.1
Setting 3	3.4	3.7	4.1	3.9	3.9	4.0
Setting 4	4.6	4.5	4.9	5.5	5.5	5.1
$m = n = 50$						
Setting 1	4.1	4.6	4.6	4.9	5.3	5.3
Setting 2	4.6	4.6	3.9	4.2	4.2	4.4
Setting 3	3.6	3.8	3.6	3.9	4.2	4.2
Setting 4	4.0	5.2	4.7	5.2	6.0	6.0
$m = n = 100$						
Setting 1	4.6	4.1	5.4	5.2	6.0	5.8
Setting 2	5.3	5.2	4.7	4.6	4.8	4.9
Setting 3	4.2	4.6	4.0	4.1	4.1	4.1
Setting 4	5.3	5.8	5.1	5.9	6.0	6.0

Table S2. Empirical Type I error rate from the simulation study with the indicators having an overall reliability of 60% and a linear relationship with the latent variable. Extreme deviations from 5% are indicated in bold.

	**Linear**					
	**WMW – max rel**	**WMW – mean**	***t* test – max rel**	***t* test – mean**	**SEM**	**SEM – corrected**
$\mathcal{N}(0,1)$						
$\boldsymbol{m = n = 15}$						
Setting 1	4.2	4.6	4.6	5.1	5.1	4.9
Setting 2	3.8	3.7	4.2	4.3	4.1	4.3
Setting 3	4.8	4.8	5.3	5.4	4.9	4.9
Setting 4	5.0	5.4	5.8	6.1	5.9	6.2
$\boldsymbol{m = n = 50}$						
Setting 1	5.1	5.2	5.8	5.0	6.3	6.1
Setting 2	5.4	5.6	6.4	6.4	**6.9**	**6.9**
Setting 3	4.4	4.0	5.4	4.9	5.6	5.6
Setting 4	6.0	4.9	5.9	5.7	6.7	6.6
$\boldsymbol{m = n = 100}$						
Setting 1	4.4	4.7	4.0	4.0	4.0	4.1
Setting 2	5.8	5.7	5.2	5.1	5.5	5.7
Setting 3	6.0	6.1	6.3	6.2	6.6	6.6
Setting 4	5.0	5.5	4.8	6.0	4.9	5.1
t_5						
$\boldsymbol{m = n = 15}$						
Setting 1	4.5	4.4	5.1	4.4	4.2	4.2
Setting 2	4.6	5.3	4.5	5.0	4.9	5.1
Setting 3	4.3	4.7	5.2	5.0	5.5	5.7
Setting 4	5.0	4.9	5.9	5.0	5.5	4.7
$\boldsymbol{m = n = 50}$						
Setting 1	5.8	5.8	5.1	4.5	5.6	5.8
Setting 2	3.4	**3.2**	3.7	3.7	4.1	4.1
Setting 3	4.6	4.7	4.3	4.5	5.4	5.1
Setting 4	4.5	4.4	4.4	4.2	4.8	5.0
$\boldsymbol{m = n = 100}$						
Setting 1	5.1	5.2	3.6	3.6	3.7	3.9
Setting 2	5.2	5.1	5.2	5.1	5.5	5.5
Setting 3	5.1	5.2	4.3	4.4	4.6	4.5
Setting 4	4.7	5.9	5.0	6.0	5.8	6.0

	**Linear**					
	**WMW – max rel**	**WMW – mean**	***t* test – max rel**	***t* test – mean**	**SEM**	**SEM – corrected**
$Laplace(0, 1.25)$						
$\boldsymbol{m = n = 15}$						
Setting 1	4.8	5.2	5.2	6.5	5.6	5.2
Setting 2	3.6	3.6	4.5	3.9	4.3	3.9
Setting 3	4.4	4.7	4.7	5.4	4.9	5.4
Setting 4	5.0	4.5	5.9	5.5	5.6	5.5
$\boldsymbol{m = n = 50}$						
Setting 1	5.5	5.5	5.1	5.5	5.6	5.7
Setting 2	5.3	5.1	5.2	5.1	6.0	6.0
Setting 3	4.8	4.9	4.9	5.2	5.6	6.0
Setting 4	5.0	4.4	5.1	4.7	5.5	5.3
$\boldsymbol{m = n = 100}$						
Setting 1	4.9	4.9	4.1	4.1	4.4	4.4
Setting 2	4.7	5.4	5.2	4.9	5.7	5.6
Setting 3	5.9	5.9	5.3	5.4	5.5	5.6
Setting 4	4.5	4.1	4.2	3.8	4.9	4.9
Exp						
$\boldsymbol{m = n = 15}$						
Setting 1	4.5	4.6	5.6	4.4	6.6	5.9
Setting 2	4.7	4.2	4.4	4.1	4.7	4.8
Setting 3	3.8	3.5	3.9	3.5	3.1	3.6
Setting 4	4.7	5.0	5.1	5.5	5.2	5.0
$\boldsymbol{m = n = 50}$						
Setting 1	3.5	4.4	4.7	4.9	5.3	4.8
Setting 2	4.3	4.6	4.2	4.4	4.6	4.8
Setting 3	3.9	4.1	4.0	4.0	4.6	4.9
Setting 4	4.3	5.3	4.6	5.4	6.1	6.1
$\boldsymbol{m = n = 100}$						
Setting 1	5.0	5.2	5.4	5.3	5.7	5.7
Setting 2	5.2	5.0	4.9	5.0	5.3	5.3
Setting 3	5.0	5.2	3.7	3.7	3.8	3.9
Setting 4	5.4	5.2	6.0	5.6	6.6	6.4

Table S3. Empirical Type I error rate from the simulation study with the indicators having an overall reliability of 80% and a non-linear relationship with the latent variable. Extreme deviations from 5% are indicated in bold.

	Non-linear					
	**WMW – max rel**	**WMW – mean**	***t* test – max rel**	***t* test – mean**	**SEM**	**SEM – corrected**
$\mathcal{N}(0,1)$						
$m = n = 15$						
Setting 1	5.0	4.8	4.6	4.8	4.7	5.3
Setting 2	4.4	4.1	4.1	4.8	4.9	4.2
Setting 3	4.2	4.6	5.3	5.4	5.3	5.3
Setting 4	5.0	5.2	5.9	5.4	6.2	6.6
$m = n = 50$						
Setting 1	4.7	4.6	4.8	4.6	5.6	5.9
Setting 2	5.3	4.8	5.7	5.8	6.3	6.4
Setting 3	4.6	4.3	4.9	5.3	5.8	5.6
Setting 4	5.6	5.3	6.6	5.2	7.1	7.1
$m = n = 100$						
Setting 1	4.0	3.5	3.9	3.6	4.2	4.1
Setting 2	6.1	6.2	5.0	4.7	5.6	5.7
Setting 3	5.5	5.5	5.9	6.0	6.3	6.3
Setting 4	5.1	6.1	3.9	5.6	4.4	4.5
t_5						
$m = n = 15$						
Setting 1	4.5	4.4	5.0	4.6	5.0	4.4
Setting 2	4.8	4.9	5.4	5.8	5.3	5.4
Setting 3	4.3	4.5	3.8	4.3	4.4	4.4
Setting 4	4.3	4.8	4.4	5.7	5.3	5.4
$m = n = 50$						
Setting 1	5.5	5.0	5.4	5.0	5.6	5.4
Setting 2	3.6	3.5	4.5	4.2	4.8	4.6
Setting 3	4.5	4.3	3.7	3.5	4.4	4.1
Setting 4	3.6	4.5	3.7	4.8	4.8	4.5
$m = n = 100$						
Setting 1	4.7	4.9	4.0	4.1	4.1	4.1
Setting 2	4.8	5.0	5.1	4.8	5.6	5.2
Setting 3	4.3	4.4	4.6	4.6	4.9	4.8
Setting 4	4.6	5.9	4.6	6.3	5.8	5.7

	Non-linear					
	**WMW – max rel**	**WMW – mean**	***t* test – max rel**	***t* test – mean**	**SEM**	**SEM – corrected**
$Laplace(0, 1.25)$						
$m = n = 15$						
Setting 1	5.0	5.1	5.1	5.2	5.5	5.5
Setting 2	3.8	3.6	4.1	4.0	5.0	4.7
Setting 3	5.0	4.9	5.6	5.5	5.2	5.3
Setting 4	4.5	4.9	5.4	4.9	5.3	5.3
$m = n = 50$						
Setting 1	5.1	5.0	6.0	5.2	6.4	6.1
Setting 2	4.3	4.4	5.3	4.6	5.6	5.8
Setting 3	4.1	4.4	5.4	5.8	6.4	6.1
Setting 4	5.1	4.5	5.1	4.2	6.1	5.6
$m = n = 100$						
Setting 1	5.3	5.1	4.7	4.6	4.8	4.6
Setting 2	4.9	5.5	5.7	5.7	5.9	5.6
Setting 3	5.4	4.8	4.9	4.7	5.0	5.0
Setting 4	4.7	4.6	3.5	4.1	4.0	4.0
Exp						
$m = n = 15$						
Setting 1	4.4	4.7	5.8	5.5	5.9	6.0
Setting 2	4.5	4.6	5.3	4.8	5.2	5.1
Setting 3	4.3	3.7	3.7	3.8	3.9	3.6
Setting 4	4.5	5.3	5.4	5.7	5.4	5.7
$m = n = 50$						
Setting 1	4.4	4.4	4.4	4.9	4.8	4.3
Setting 2	5.2	4.5	4.9	4.8	5.5	4.9
Setting 3	3.6	4.0	3.5	3.5	4.6	3.8
Setting 4	4.6	4.7	4.8	5.0	6.4	5.9
$m = n = 100$						
Setting 1	4.6	4.4	4.9	4.8	5.1	4.8
Setting 2	5.3	5.3	5.5	5.4	5.9	5.5
Setting 3	4.3	4.1	4.4	4.4	4.5	4.4
Setting 4	5.2	5.8	5.3	5.8	6.0	5.7

Table S4. Empirical Type I error rate from the simulation study with the indicators having an overall reliability of 60% and a non-linear relationship with the latent variable. Extreme deviations from 5% are indicated in bold.

	Non-linear					
	**WMW – max rel**	**WMW – mean**	***t* test – max rel**	***t* test – mean**	**SEM**	**SEM – corrected**
$\mathcal{N}(0,1)$						
$m = n = 15$						
Setting 1	4.2	4.5	4.8	5.1	5.5	4.9
Setting 2	3.4	4.2	3.6	4.0	4.2	4.2
Setting 3	5.0	5.0	5.3	5.3	5.3	5.2
Setting 4	4.2	5.2	5.4	5.9	6.0	6.7
$m = n = 50$						
Setting 1	5.5	5.4	5.5	5.2	6.1	6.2
Setting 2	5.4	5.6	6.3	6.5	6.2	6.4
Setting 3	4.4	4.2	4.7	5.0	5.1	5.5
Setting 4	5.8	5.1	6.1	5.6	6.4	6.3
$m = n = 100$						
Setting 1	4.4	4.4	4.1	4.4	4.2	4.1
Setting 2	5.9	5.8	5.5	5.2	5.6	5.6
Setting 3	6.0	6.3	6.4	6.2	6.7	6.6
Setting 4	5.0	5.5	4.4	5.5	4.6	4.9
t_5						
$m = n = 15$						
Setting 1	4.2	4.2	5.3	4.7	5.0	4.4
Setting 2	5.2	5.1	5.3	5.7	5.3	5.2
Setting 3	4.1	4.3	4.8	4.6	5.3	5.2
Setting 4	4.8	5.5	5.5	5.5	5.4	4.7
$m = n = 50$						
Setting 1	5.7	5.7	6.0	4.9	6.6	6.5
Setting 2	3.5	**3.0**	4.1	4.0	4.6	4.0
Setting 3	4.4	4.2	3.9	3.6	4.3	4.1
Setting 4	4.3	4.5	4.3	4.6	4.6	4.6
$m = n = 100$						
Setting 1	5.1	4.8	4.0	4.0	4.3	4.1
Setting 2	5.0	5.4	4.9	5.5	5.6	5.5
Setting 3	5.3	5.0	4.5	4.6	4.5	4.5
Setting 4	5.2	5.8	5.0	6.2	5.7	5.6

	Non-linear					
	**WMW – max rel**	**WMW – mean**	***t* test – max rel**	***t* test – mean**	**SEM**	**SEM – corrected**
$Laplace(0,1.25)$						
$m = n = 15$						
Setting 1	4.7	5.7	4.7	5.9	5.3	5.1
Setting 2	3.9	3.3	4.3	3.7	4.5	4.2
Setting 3	4.4	5.2	4.9	5.1	4.9	5.8
Setting 4	4.4	4.4	5.8	5.3	5.4	5.8
$m = n = 50$						
Setting 1	5.3	5.1	5.0	5.4	5.6	5.7
Setting 2	5.4	4.8	5.1	5.2	5.8	5.8
Setting 3	4.7	5.2	5.4	5.6	5.8	6.0
Setting 4	4.8	4.4	4.9	4.3	5.5	5.6
$m = n = 100$						
Setting 1	4.7	4.6	4.7	4.6	4.8	4.7
Setting 2	4.7	5.0	5.2	5.2	5.6	5.4
Setting 3	5.5	5.7	5.2	5.2	5.6	5.5
Setting 4	4.4	4.4	4.1	4.2	4.9	4.7
Exp						
$m = n = 15$						
Setting 1	4.4	4.9	5.2	4.8	5.9	6.2
Setting 2	4.5	4.1	4.7	4.3	5.0	5.0
Setting 3	3.8	3.3	4.2	3.6	3.7	3.4
Setting 4	4.5	5.2	4.9	5.3	5.1	5.0
$m = n = 50$						
Setting 1	4.2	4.7	4.2	4.6	4.6	4.0
Setting 2	5.1	4.7	5.2	5.0	5.5	5.2
Setting 3	3.6	4.2	3.9	4.2	4.5	3.8
Setting 4	4.8	5.4	5.2	5.4	5.8	5.6
$m = n = 100$						
Setting 1	5.0	4.4	5.2	5.3	5.4	5.0
Setting 2	5.6	5.2	5.3	5.5	5.5	5.2
Setting 3	4.3	4.7	3.6	3.8	4.0	3.9
Setting 4	5.5	5.1	5.3	5.3	6.1	5.8

2 VISUAL INSPECTION Q–Q PLOTS LOCATION SHIFT

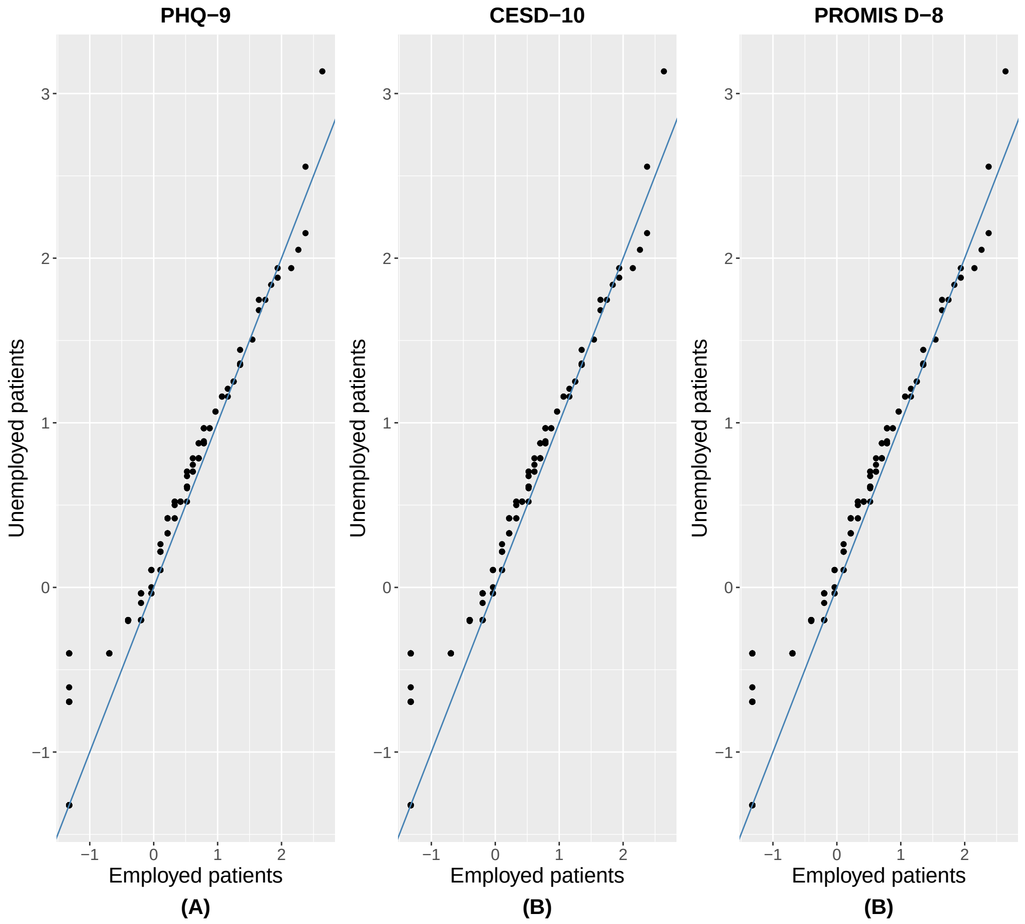

Figure S1. Q–Q plots for **(A)** the Patient Health Questionnaire-9 (PHQ-9), **(B)** the Center for Epidemiological Studies Depression Scale-10 (CESD-10) and **(C)** the eight-item PROMIS Depression Short Form (PROMIS D-8). A visual inspection shows that the assumption of location shift for the indicators is reasonably met.
